# TGF-β Signalling Is Required for CD4^+^ T Cell Homeostasis But Dispensable for Regulatory T Cell Function

**DOI:** 10.1371/journal.pbio.1001674

**Published:** 2013-10-08

**Authors:** Anna Śledzińska, Saskia Hemmers, Florian Mair, Oliver Gorka, Jürgen Ruland, Lynsey Fairbairn, Anja Nissler, Werner Müller, Ari Waisman, Burkhard Becher, Thorsten Buch

**Affiliations:** 1 Institute of Experimental Immunology, University of Zurich, Zurich, Switzerland; 2 Institute for Genetics, University of Cologne, Cologne, Germany; 3 Clinical Chemistry, Klinikum rechts der Isar, Technische Universität München, Germany; 4 Institute for Medical Microbiology, Immunology and Hygiene, Technische Universität München, Germany; 5 Department of Experimental Immunology, Helmholtz Center for Infection Research, Braunschweig, Germany; 6 Institute for Molecular Medicine, University Medical Center of the Johannes-Gutenberg University of Mainz, Mainz, Germany; National Jewish Medical and Research Center/Howard Hughes Medical Institute, United States of America

## Abstract

TGF-β is widely held to be critical for the maintenance and function of regulatory T (T_reg_) cells and thus peripheral tolerance. This is highlighted by constitutive ablation of TGF-β receptor (TR) during thymic development in mice, which leads to a lethal autoimmune syndrome. Here we describe that TGF-β–driven peripheral tolerance is not regulated by TGF-β signalling on mature CD4^+^ T cells. Inducible TR2 ablation specifically on CD4^+^ T cells did not result in a lethal autoinflammation. Transfer of these TR2-deficient CD4^+^ T cells to lymphopenic recipients resulted in colitis, but not overt autoimmunity. In contrast, thymic ablation of TR2 in combination with lymphopenia led to lethal multi-organ inflammation. Interestingly, deletion of TR2 on mature CD4^+^ T cells does not result in the collapse of the T_reg_ cell population as observed in constitutive models. Instead, a pronounced enlargement of both regulatory and effector memory T cell pools was observed. This expansion is cell-intrinsic and seems to be caused by increased T cell receptor sensitivity independently of common gamma chain-dependent cytokine signals. The expression of Foxp3 and other regulatory T cells markers was not dependent on TGF-β signalling and the TR2–deficient T_reg_ cells retained their suppressive function both *in vitro* and *in vivo*. In summary, absence of TGF-β signalling on mature CD4^+^ T cells is not responsible for breakdown of peripheral tolerance, but rather controls homeostasis of mature T cells in adult mice.

## Abstract 2

Signalling by the cytokine TGF-β regulates mature CD4+ T cell populations but is not involved in the survival and function of regulatory T cells.

## Author Summary

TGF-β is a cytokine thought to be critical for the maintenance and function of tolerance in the immune system. In many studies the disruption of TGF-β signalling in CD4^+^ T cells (a type of white blood cell that coordinates immune responses) has resulted in autoimmune syndromes. We show here that the induced removal of this cytokine's receptor from these specialised blood cells results in an astonishingly mild outcome. Contrary to expectations, the number of regulatory T cells is actually increased, and we find that these cells are not dependent on TGF-β signalling. We also show that removal of the receptor from mature CD4^+^ T cells does not lead to lethal autoinflammation; only when we removed the receptor during development of the cells did we see the characteristic lethal multi-organ inflammation reported previously in constitutive models of TGF-β receptor ablation. In summary, our findings indicate that although TGF-β regulates maintenance of mature CD4^+^ T cells, its signals are dispensable for immune tolerance within this cell population.

## Introduction

Transforming growth factor β (TGF-β1) is a cytokine that is expressed throughout the hematopoietic system. It was initially described to suppress T cell proliferation [1], but later found to also support differentiation of T cells into specialized subsets. Mice deficient in TGF-β1 show hyperactivation and uncontrolled expansion of T cells leading to a lethal multi-organ autoimmune disorder [2]. Likewise, deficiency of either subunit of the heterodimeric TGF-β receptor (TR) is lethal [3],[4]. T-cell–specific expression of a dominant-negative receptor mutant (DN-TR2) under the CD4 promoter results in a lymphoproliferative disease with infiltrates to multiple organs and development of severe inflammatory bowel disease [5]. Similar mice that express a DN-TR2 construct under human CD2 promoter presented with disease symptoms at the age of 3–4 mo [6]. The lymphoproliferative disease in these mice is caused primarily by expansion of CD8^+^ T cells that even progress to leukaemia [7],[8]. Genetic ablation of TR2 in hematopoietic or, more specifically, in T cells leads to lymphadenopathy, splenomegaly, and inflammation of various organs followed by death at 3 wk of age [9]–[11]. Thus, all these models of interrupted TR function suggest an essential, nonredundant role for TGF-β1 in the maintenance of T cell tolerance. The observed autoimmune syndrome was initially thought to be caused by the apparent loss of regulatory T (T_reg_) cells. Particularly thymic T_reg_ development and survival seem to require TGF-β signals [10]–[12]. Yet neither reconstitution with WT T_reg_ cells nor mixed bone marrow chimera experiments resulted in an amelioration of the autoimmune phenotype in these models [10],[11]. Notwithstanding that cell type and reason for T-cell–mediated autoimmunity in the absence of TGF-β signalling remain unclear, in all these models thymic development was found to be altered [11]–[14]. A recent study shows that deletion of TR2 in mature T cells by dLck-Cre does not cause a fatal autoimmune syndrome [15]. In this model, however, the role of TGF-β signalling in mature T_reg_ cells could not be addressed, since dLck-Cre did not lead to deletion of TR2 in Foxp3^+^ CD4^+^ T cells. Thus, our current, unbiased knowledge about the *in vivo* role of TGF-β for peripheral T, especially T_reg_, cells appears to be incomplete. To overcome this and analyze TGF-β function in T helper and T_reg_ cells independent of developmental defects as well as systemic autoimmunity, we inducibly abrogated TGF-β signalling in peripheral CD4^+^ T cells. Surprisingly, loss of TR2 function in mature T cells, including T_reg_ cells, did not lead to the spontaneous development of autoimmunity. Adoptive transfer of TR2-deficient CD4^+^ T cells into lymphopenic hosts led only to colitis but not systemic disease. However, the induced TR2 deletion in thymocytes of lymphopenic mice resulted in a rapidly developing lethal auto-inflammatory disorder. When TR2 ablation was restricted to postthymic T cells, we observed that not only T_em_ (CD62L^lo^CD44^hi^) cells but also T_reg_ cells exhibited hyperproliferation resulting from increased sensitivity to TCR signalling. TR2-deficient T_reg_ cells retained their suppressive capacity both *in vitro* and *in vivo*. For mature CD4^+^ T cells in the adult mouse, TGF-β seems therefore to solely be a regulator of T_reg_ as well as T_em_ cell expansion but is not required for the maintenance of tolerance.

## Results

### Induced Ablation of TR2 From Peripheral CD4^+^ T Cells

**Figure 1:**
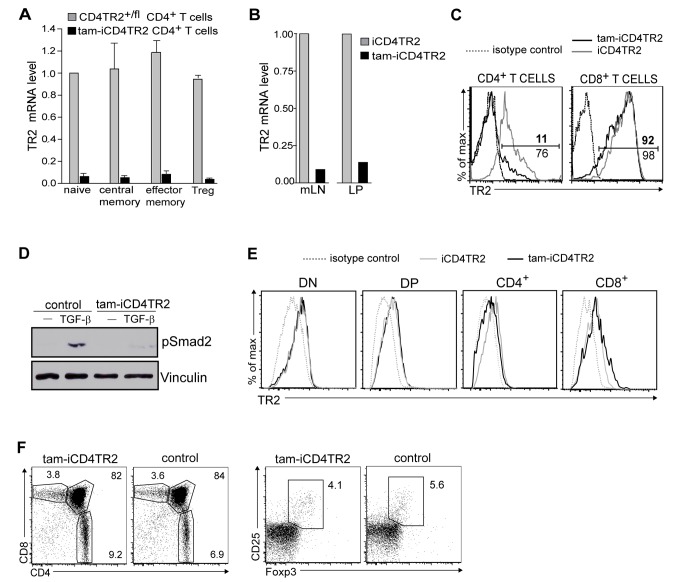
Validation of CD4-CreER^t2^/tamoxifen-mediated TR2 ablation. (A) Quantitative RT-PCR of TR2 mRNA in FACS-sorted splenic CD4^+^ T cell subsets. These data are representative results of three independent experiments. (B) Quantitative RT-PCR of TR2 mRNA in FACS-sorted CD4^+^ T cells from mesenteric lymph nodes and lamina propria. These data are representative results of three independent experiments. (C) Flow cytometric analysis of TR2 expression by CD4^+^ and CD8^+^ T cells from peripheral blood. These data are representative results of four independent experiments. (D) Western blot analysis of pSmad2 and Vinculin in lysates of magnetically purified splenic CD4^+^ T cells from tam-iCD4TR2 and control mice 2 wk p.a.. The cells were cultured for 40 min in the presence of antiCD3/CD28 either with or without 20 ng/ml TGFβ-1. These are representative data of two independent experiments. (E) Flow cytometric analysis of TR2 expression by thymocytes 2 wk p.a. These data are representative results of two independent experiments. (F) Flow cytometric analysis of expression of CD4 and CD8 by thymocytes (left panel) as well as Foxp3 and CD25 by CD4^+^ SP thymocytes (right panel) from tam-iCD4TR2 and control mice at 2 wk p.a. These data are representative results of three independent experiments.

Previous genetic studies of the function of TGF-β1 in T cells have either relied on germline gene deficiencies or involved cell-type–specific mutagenesis. Consequently, these models were biased by strong alterations in thymic development [10]–[13]. To specifically address the role of TGF-β signalling in mature CD4^+^ T cells, we circumvented any developmental impact through the generation and use of a new tamoxifen-inducible [16] CD4-CreER^t2^ knock-in strain (Figure S1A,B). We first tested the tamoxifen-induced recombination of this strain with the help of two fluorescent reporter mouse strains (RAGE and ROSA-EYFP) and found efficient and specific recombination in CD4^+^ T cells (unpublished data). As anticipated, recombination also took place in a small fraction of CD4^+^ splenic dendritic cells (DCs) and innate lymphoid cells (unpublished data). Since removal of the neomycin resistance gene (Neo(R)) by FLP-mediated deletion [17] reduced recombination efficiency without gain of specificity, a phenomenon observed before [18], we used animals bearing the Neo(R)-containing allele for all further experiments. To investigate the role of TGF-β signalling in peripheral T helper cells, we crossed the CD4-CreER^t2^ with a conditional TR2 allele (TR2^f^) [9] obtaining CD4-CreER^t2^/TR2^f/f^ (hereafter called iCD4TR2) experimental mice as well as CD4-CreER^t2^/TR2^f/+^ and TR2^f/f^ control mice. First, we tested ablation of TR2 in CD4^+^ T cells 14 d after commencing 5 d of tamoxifen treatment of iCD4TR2 mice (hereafter called tam-iCD4TR2 mice). mRNA levels within sorted naïve, central memory, effector memory/effector (T_em_) and regulatory (T_reg_) CD4^+^ T cells from tam-iCD4TR2 mice indicated a recombination frequency of 90%–95% in mature T cells (Figure 1A), placing it at similar efficiency as many constitutive Cre strains [10],[11]. Equally high deletion efficiencies were observed in CD4^+^ T cells from mesenteric LN (mLN) and the lamina propria (LP) (Figure 1B). Surface expression analysis of TR2 on blood and spleen CD4^+^ T cells supported this observation (Figure 1C, Figure S2A, and unpublished data) and also showed that expression of TR2 by other cell types including CD8^+^ T cells was unaffected (Figure 1C and Figure S2A). As expected, TGF-β–induced phosphorylation of Smad2 [19] was virtually absent in CD4^+^ T cells from tam-iCD4TR2 mice 2 wk p.a. (Figure 1D). We thus conclude that target allele recombination in our inducible system is specific and efficient.

Because CD4-Cre-mediated TR2-ablation resulted in a strong thymic phenotype including reduction of single positive CD8^+^ T cells, abrogation of NK T cell development, and enhanced negative selection [11],[12], we investigated the extent and consequences of induced TR2 ablation in the thymus. Two weeks p.a. the surface expression of TR2 was reduced only slightly on CD4^+^ thymocytes but not on any of the other major thymocyte compartments (Figure 1E). Reduction of TR2 mRNA levels was found not only in CD4^+^ but also in a small fraction of CD8^+^ SP thymocytes only 2 wk p.a., indicating low-level and transient recombination at the DP stage (Figure S2B). Also, expression of CD4, CD8, CD5, CD24, CD62L, and CD69 were unchanged in the thymus (Figure 1F and unpublished data) as were number and phenotype of thymic T_reg_ cells (Figure 1F). Thus, our model allows the study of TGF-β signalling in mature CD4^+^ T cells by pulse-chase experiments without significantly disturbing thymic T cell development.

### Peripheral Abrogation of TR2 Signalling in CD4^+^ T Cells Does Not Lead to Autoimmunity

**Figure 2:**
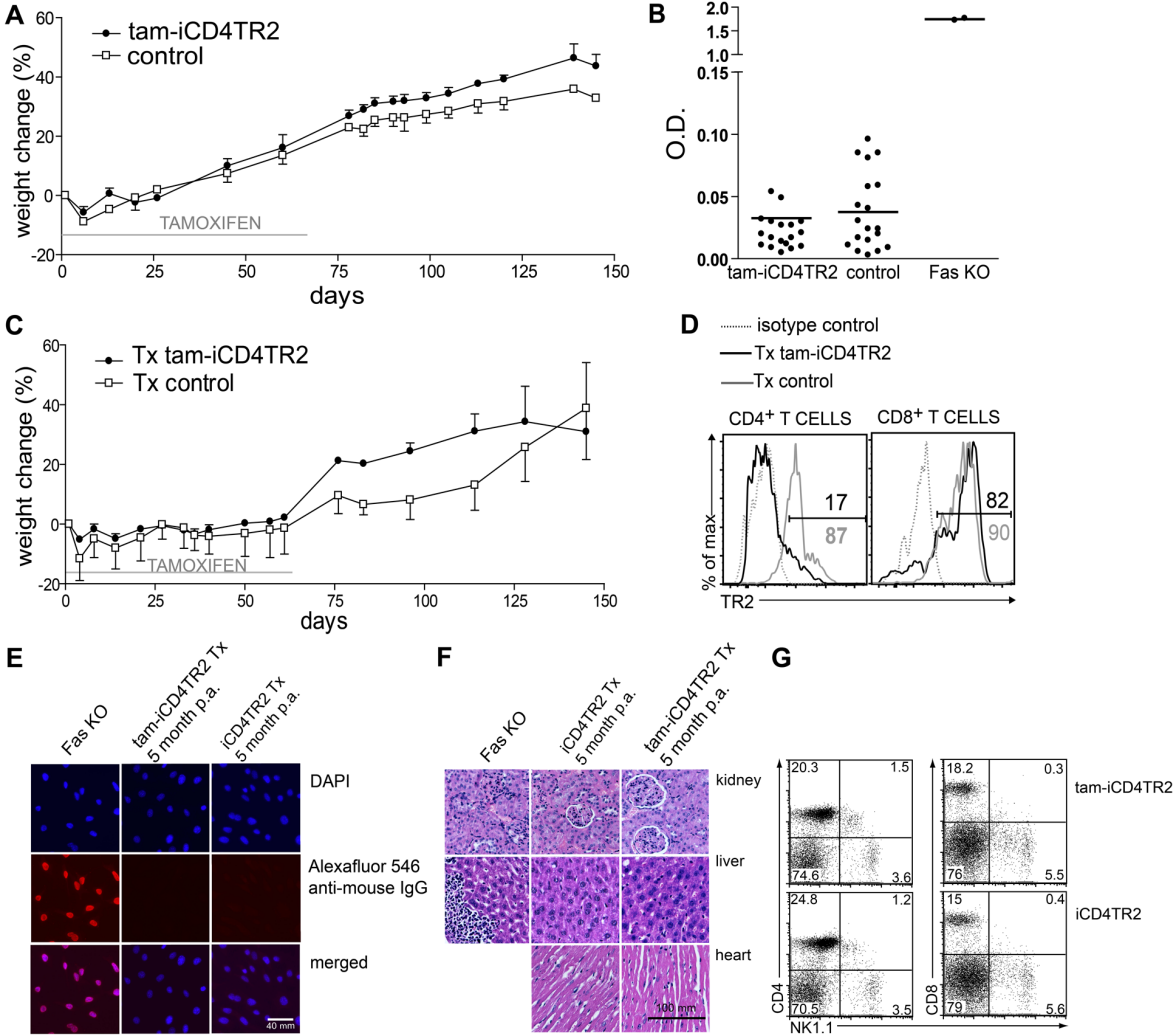
Absence of autoimmunity after TR2 ablation in mature CD4^+^ T cells. (A) iCD4TR2 and control mice were treated with tamoxifen citrate for 2 mo followed by 3 mo on normal diet and body weight was monitored (mean ± SEM, 5 mice per group, representative data of two independent experiments). (B) ELISA for anti-dsDNA antibodies in sera from tam-iCD4TR2 and control mice 2, 4, 6 wk, and 5 month p.a.; as positive control a serum from a *fas*-deficient mouse was used. (C) iCD4TR2 and control mice were thymectomised 1 wk before the beginning of tamoxifen treatment and treated with tamoxifen citrate for 2 mo followed by 3 mo on normal diet and body weight was monitored (mean ± SEM, 5 mice per group, representative data of two independent experiments). (D) Flow cytometric analysis of TR2 expression by CD4^+^ and CD8^+^ T cells from peripheral blood after 5 mo postthymectomy and tamoxifen treatment. (E) Analysis of antinuclear antibodies by immunofluorescent staining of NIH3T3 cells with sera from indicated mice. Representative micrographs are shown. The size bar indicates 40 µm. (F) Representative micrographs of H&E-stained tissue sections of indicated organs isolated from thymectomised tam-iCD4TR2 or control mice from (C). The size bar indicates 100 µm. (G) Flow cytometric analysis of NK1.1 expression on splenic CD4^+^ and CD8^+^ T cells isolated from tam-iCD4TR2 or control mice 2 wk posttamoxifen administration. These are representative data of three independent experiments.

Constitutive ablation of TGF-β signalling during thymic development of αβT cells (CD4-Cre or Lck-Cre–mediated) but not during peripheral life (dLck-Cre–mediated) invariably results in a generalised and rapidly lethal autoimmune disorder [10],[11],[14],[15]. When we followed tam-iCD4TR2 mice after induction of receptor deletion, they appeared healthy and showed no weight loss 2 and 4 wk after 5 d of tamoxifen treatment and up to 3 mo after feeding with tamoxifen citrate for 2 mo (Figure 2A). Because mild autoimmunity may not present clinically, we performed histopathological analysis of liver, kidney, pancreas, heart, colon, and thyroid gland but could not detect cellular infiltrates in any of these organs (unpublished data). We also tested for secondary dysregulation of B cell tolerance manifested by autoantibody production, as previously described upon thymic deletion of TR2 expression [11]. Yet sera from tam-iCD4TR2 mice at 6 wk or even 5 mo p.a. did not contain significant levels of autoantibodies as shown by ELISA against dsDNA (Figure 2B). Thus, removal of TR2 from mature CD4^+^ T cells does not result in tolerance loss or autoimmunity. To exclude that recent thymic emigrants could dilute out cells lacking TR2, we thymectomized mice 1 wk before an 8-wk tamoxifen citrate treatment. Again, we failed to observe any signs of autoimmune disease by clinical appearance and weight loss up to 5 mo p.a. (Figure 2C) even though the frequency of TR2-deficient cells remained constant until the end of the experiment (Figure 2D). The absence of elevated serum antibody levels, autoantibodies, and organ infiltrates again excluded subclinical tolerance loss (Figure 2E,F and unpublished data). Furthermore, CD8^low^ or CD4^+^ NK1.1^+^ T cells, proposed to carry autoreactive activity in the constitutive TR2-deficient models [10], were not detected 2 and 4 wk as well as 5 mo p.a. and after thymectomy (Figure 2G and unpublished data). In conclusion, in contrast to models of constitutively impaired TGF-β signalling in T cells [5],[6],[10],[11], induced TR2 ablation from peripheral CD4^+^ T cells does not result in the loss of self-tolerance.

### Induced Ablation of TR2 During Thymic Development Combined with Lymphopenia Results in Lethal Autoimmunity

**Figure 3:**
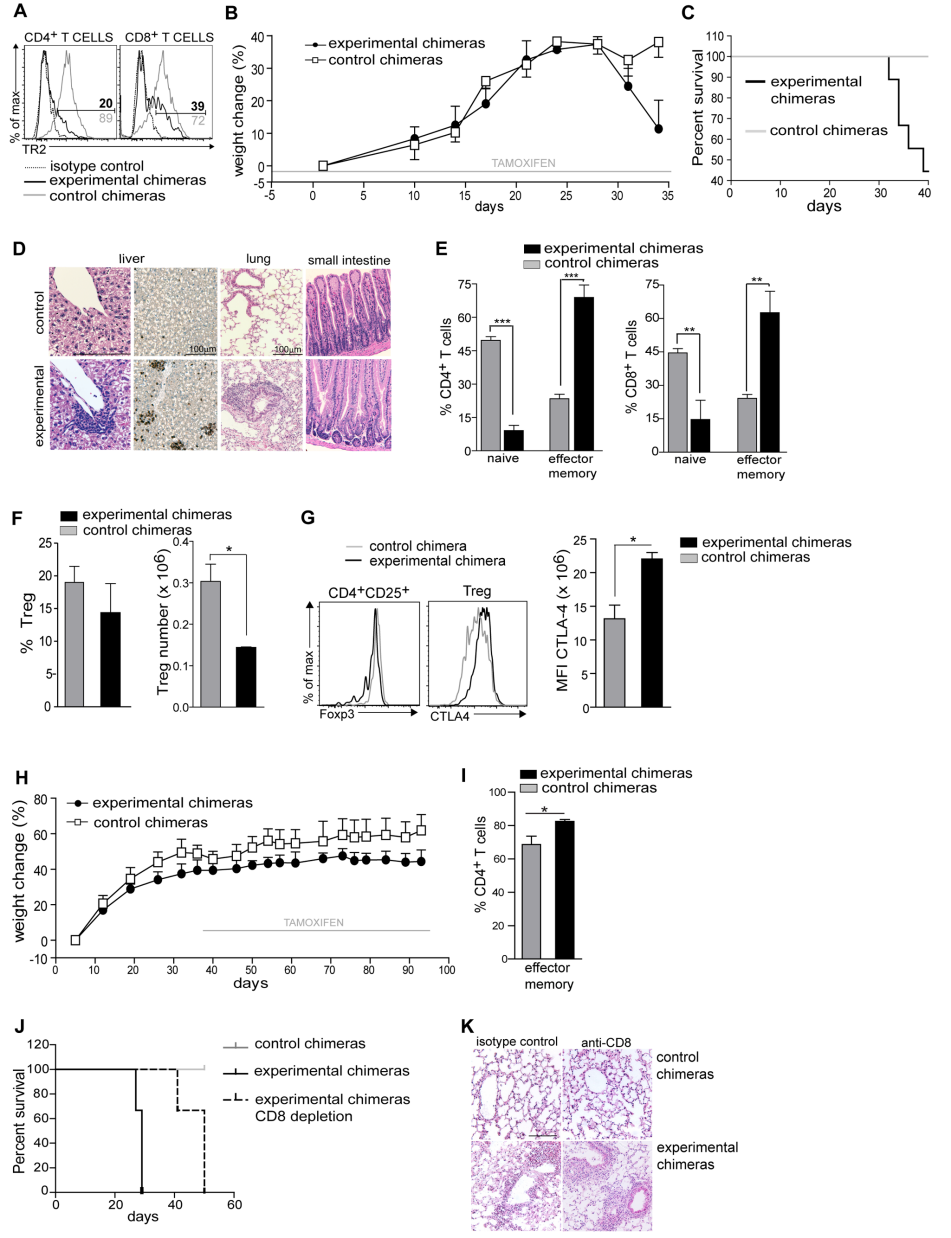
Development of lethal autoimmunity after thymic deletion of TR2. Tamoxifen-treatment of Rag1^−/−^ mice started 3 d before reconstitution with T-cell–depleted bone marrow from iCD4TR2 or control mice (A–G). (A) Flow cytometric analysis of TR2 expression by CD4^+^ and CD8^+^ T cells at day 34. Representative data of two independent experiments. (B) Body weight was monitored during the whole experiment (mean ± SEM, 5 mice per group, representative data of two independent experiments). (C) Kaplan-Meyer survival graph for all animals of experiments. (D) Representative micrographs of H&E- and anti-CD3-stained tissue sections of indicated organs at day 34. The size bar indicates 100 µm. (E) Flow cytometric analysis of the expression of CD44 and CD62l by CD4^+^ and CD8^+^ T cells. The percentage of T_n_ and T_em_ cells in the spleen of experimental and control chimeric mice (mean ± SEM, 6 mice per group, analysed in two independent experiments). (F) Percentage and number of splenic T_reg_ cells at day 34 (mean ± SEM, 5 mice per group; representative data of two independent experiments). (G) Flow cytometric analysis of FoxP3 and CTLA-4 by indicated splenic CD4^+^ T cells subsets at day 34 (representative data of three independent experiments). Mean florescence intensity of CTLA-4 expression by splenic T_reg_ cells (right panel, mean ± SEM, 4 mice per group; representative data of three independent experiments). (H) Rag1^−/−^ mice were reconstituted with T-cell–depleted bone marrow from iCD4TR2 or control mice. Tamoxifen treatment of recipients started 5 wk postreconstitution and body weight was monitored during the whole experiment (mean ± SEM, 5 mice per group; representative data of two independent experiments). (I) The percentage of T_em_, cells in the spleen of experimental and control chimeric mice (mean ± SEM, 6 mice per group, analysed in two independent experiments). (J) Tamoxifen-treatment of Rag1^−/−^ mice started 3 d before reconstitution with T-cell–depleted bone marrow from iCD4TR2 or control mice. At day 15 posttransfer treatment with anti-CD8 antibody or isotype control started. Shown is a Kaplan-Meyer survival graph for all animals in the experiments (representative data of two independent experiments). (K) Representative micrographs of H&E-stained lung sections in the terminal stage of the disease. The size bar indicates 100 µm.

Abrogation of TGF-β signalling through CD4-Cre takes place during thymic development and affects all αβT cells including T_reg_ cells. This thymic TR deletion results in lethal autoimmunity, in contrast to peripheral TR deletion through CD4-CreER^t2^. To understand the underlying cause of the different outcomes of TR2 ablation in T cells, we assessed whether they were caused by deletion frequency, by ablation during thymic development, or by lymphopenia. To mimic the environment found in neonates with constitutive T-cell–specific TR2 ablation [10],[11], we induced recombination during thymic development in a lymphopenic environment: We initiated treatment with tamoxifen of *Rag-1* ^−/−^ mice 3 d before irradiation and reconstitution with bone marrow of iCD4TR2 or control mice (experimental scheme outlined in Figure S3A). Tamoxifen treatment led to deletion of TR2 in the thymus of mice reconstituted with iCD4TR2 bone marrow: both CD4^+^ and CD8^+^ T cells lacked TR2 expression (Figure 3A). The mice developed signs of sickness 28 d after bone marrow reconstitution (apathy, runted appearance, weight loss) and started dying after 30 d (Figure 3B,C). Mice that received control bone marrow (CD4-CreER^t2^/TR2^f/+^) did not shown any signs of disease (Figure 3B). Sick bone marrow chimeras presented with T cell infiltrates only in lung and liver tissue but not the colon (Figure 3D and unpublished data). The frequency of CD62L^hi^CD44^−^ naïve T (T_n_) cells was severely reduced and of CD62L^lo^CD44^+^ T_em_ cells highly increased in both CD4^+^ and CD8^+^ T cell compartments (Figure 3E). As has been reported before in constitutive CD4-Cre–mediated deletion [10],[11], we also observed a significantly decreased number of peripheral T_reg_ cells (Figure 3F). CD4^+^CD25^high^ T cells showed similar levels of Foxp3 expression while CTLA-4 was significantly up-regulated (Figure 3G). When tamoxifen treatment started 5 wk after bone marrow transfer, we observed no sign of autoimmunity, tissue infiltration, and only slight T_em_ expansion (Figure 3H,I, Figure S3B, and unpublished data), thus excluding the possibility that bone marrow chimerism per se could predispose to development of the disease.

The treatment of bone marrow chimeras with tamoxifen from the onset on led to deletion of TR2 also in CD8^+^ T cells, indicating efficient recombination already during the thymic DP stage. To exclude that these TR2-negative CD8^+^ T cells were causing disease, we repeated the experiment described in Figure 3A–G with deletion of CD8^+^ T cells (Figure S3C). Experimental chimeras treated with anti-CD8 antibody developed the lethal autoimmune syndrome only slightly later than those treated with isotype control (Figure 3J). Lungs and livers isolated from both groups of experimental chimeras presented with infiltrates (Figure 3K and unpublished data). We found no difference in weight loss, survival, or tissue pathology between control mice treated with anti-CD8 antibody or isotype control. Also, TR2 deletion was equally efficient in both groups (unpublished data). Thus, CD4^+^ T cells are the main effectors of disease upon TR2 ablation during thymic development in a lymphopenic situation.

Taken together, the fact that the TR2 deletion frequency in CD4^+^ T cells is similar in all these bone marrow chimerism experiments suggests that the cause of the autoimmune syndrome is either the absence of TR2 during thymic development or during repopulation of a lymphopenic environment, thus mimicking the observations made in constitutive models of TR2 deletion (i.e., CD4-Cre/TR2^f/f^).

### Transfer of TR2-Deficient CD4^+^ T Cells Into Lymphopenic Hosts Does Not Lead to Multi-Organ Inflammation But to Colitis

**Figure 4:**
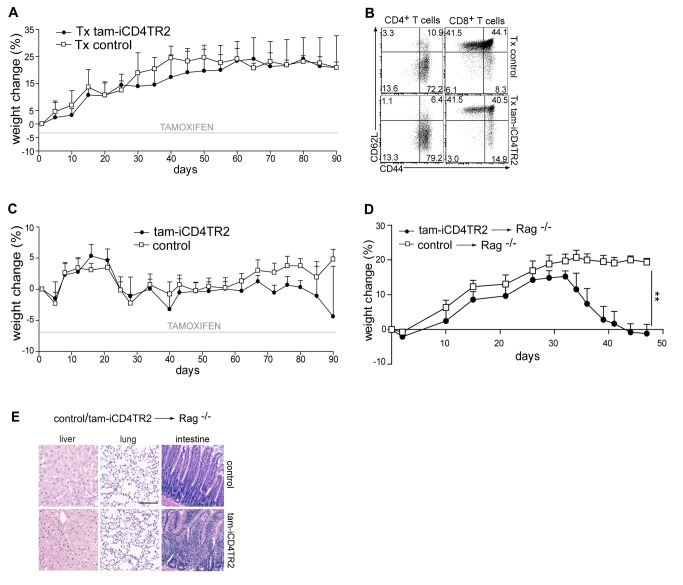
TR2-deficiency in CD4^+^ T cells in combination with severe lymphopenia leads to colitis. (A) iCD4TR2 and control mice were thymectomised at the age of 7 wk, 1 wk before tamoxifen treatment started, and treated once (day 0) with anti-CD4 antibody. Body weight was monitored during whole period of experiment (mean ± SEM, 4 mice per group). (B) Flow cytometric analysis of CD44 and CD62l expression by splenic CD4^+^ and CD8^+^ T cells isolated at day 90 (experiment as in A). (C) iCD4TR2 and control mice were irradiated (550 rad) 5 d after starting tamoxifen treatment. Tamoxifen treatment was continued for 90 d. Body weight was monitored during the whole period of experiment (mean ± SEM, 4 mice per group). (D) Purified T cells isolated from tam-iCD4TR2 and control mice 2 wk p.a. were transferred to Rag^−/−^ mice. Body weight was monitored during the whole period of the experiment (mean ± SEM, 5 mice per group). (E) Representative micrographs of H&E-stained tissue sections of indicated organs isolated from Rag^−/−^ mice 7 wk after adoptive transfer of T cells. The size bar indicates 200 µm.

To distinguish between altered thymic development and lymphopenia as cause of the autoinflammation, we assessed the influence that different lymphopenic conditions had on the behaviour of TR2-deficient CD4^+^ T cells. To achieve CD4^+^ T-cell–restricted lymphopenia, we treated thymectomized animals with a low dose of anti-CD4 antibody. This treatment led to a ∼90% depletion of blood CD4^+^ T cells (Figure 4A, Figure S4A, and unpublished data) followed by acquisition of an activated phenotype by the remaining CD4^+^ T cells in both control and experimental mice (Figure 4B). To obtain severe general lymphopenia we used sublethal irradiation (Figure 4C and Figure S4B). In this setting both CD4^+^ and CD8^+^ T cells acquired the activated phenotype (unpublished data). Even though in both acute lymphopenic conditions we observed pronounced lymphopenia-driven T cell expansion, none of the animals presented with signs of autoimmune disease by appearance, anti-dsDNA autoantibody titres, and histology until week 20 (unpublished data). Again, TR2 ablation was similarly efficient throughout these experiments (Figure S4A). Only when we adoptively transferred TR2-deficient cells into a completely lymphopenic environment (RAG-1 deficiency), strong colitis developed within 30 d (Figure 4D,E and unpublished data), which was accompanied by a massive infiltration of CD4^+^ T cells to mesenteric lymph nodes (Figure S4C). Interestingly, the mice presented with neither apathy nor runted appearance, and histology did not reveal any inflammation of lungs or livers, which is a characteristic feature in mice in which TGF-β signalling is abrogated in thymocytes (Figure 4E and unpublished data). Thus, adoptive transfer-mediated colitis can be induced by TR2-deficient T cells even in the presence of T_reg_ cells, but a general autoinflammatory syndrome is not observed. *In vivo* TR2 deletion in CD4^+^ T cells combined with acute lymphopenia, however, does not lead to loss of tolerance.

### Dysregulated Effector CD4^+^ T Cell Homeostasis in Absence of TGF-β Signalling

**Figure 5:**
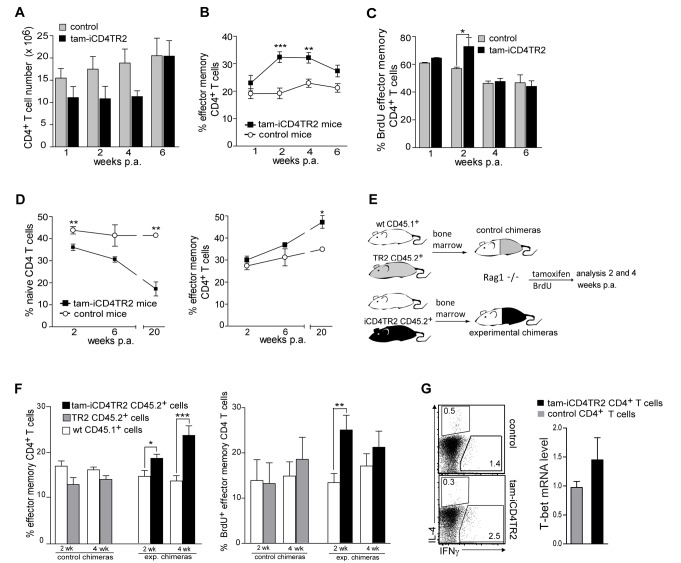
Increased proliferation of T_em_ cells upon removal of TR2. (A) Absolute number of CD4^+^ T cells in spleens of tam-iCD4TR2 and control mice 1, 2, 4, and 6 wk p.a. Mice were treated with tamoxifen for 5 consecutive days (mean ± SEM, 9 mice per group, analysed in three independent experiments). (B) Percentage of T_em_, cells in the spleen of tam-iCD4TR2, and control mice at indicated time points (percentage out of CD4^+^ T cell, mean ± SEM, 9 mice per group, analysed in three independent experiments). (C) The percentages of BrdU^+^ T_em_ cells isolated from spleens of tam-iCD4TR2 and control mice, gated on CD4^+^ T cells (mean ± SEM, 9 mice per group, analysed in three independent experiments). (D) The percentage of T_n_ and T_em_ cells in the spleen of thymectomised tam-iCD4TR2 and control mice at indicated time points (mean ± SEM, 9 mice per group, analysed in two independent experiments). (E) Rag1^−/−^ mice were reconstituted with T-cell–depleted bone marrow from WT CD45.1^+^ and CD45.2^+^ iCD4TR2 or TR2 mice in 1∶1 ratio and treated with tamoxifen for 5 consecutive days 5 wk postreconstitution. Scheme of the experimental setup. (F) The percentage of CD4^+^ T_em_ of total CD4^+^ T cells from LNs are shown (left panel) and the percentage of BrdU^+^ T_em_ cells isolated from LNs (right panel) (mean ± SEM, 10 mice per group, analysed in three independent experiments). (G) Flow cytometric analysis of the expression of IFN-γ and IL-4 by splenic CD4^+^ T cells from tam-iCD4TR2 and control mice 2 wk p.a. (representative data of two independent experiments). Quantitative RT-PCR of T-bet mRNA in sorted splenic CD4^+^ T cell. These data are representative results of two independent experiments (right panel).

To better understand the role of TGF-β signalling in mature CD4^+^ T cells, we analysed T effector homeostasis after TR2 removal in a longitudinal manner. We found slightly reduced CD4^+^ T cell numbers in spleen and LNs 2 and 4 wk p.a. (Figure 5A and unpublished data) while the total number of CD8^+^ and of central memory CD4^+^ T (CD62l^hi^CD44^+^) cells remained unchanged (unpublished data). In addition, we observed a modest but significant expansion of T_em_ cells. This phenotype was transient as cell numbers and the frequency of T_em_ cells returned to normal 6 wk p.a. (Figure 5B and unpublished data). In support of this observation, BrdU incorporation revealed increased proliferation of T_em_ but not of T_n_ and central memory CD4^+^ T cells 2 wk p.a. (Figure 5C and unpublished data). To test whether the increase of T_em_ cells was transient due to replacement by new TR2-expressing T cells, we thymectomized mice prior to TR2 ablation. In the absence of thymic emigration we observed that the elevated numbers of T_em_ cells persisted (Figure 5D).

To investigate whether the activation and proliferation of T_em_ cells upon TR2 ablation was a cell-intrinsic property or driven in trans by cell extrinsic factors, we generated bone marrow chimeras by mixing WT CD45.1^+^ and either iCD4TR2 CD45.2^+^ or control TR2^f/f^ CD45.2^+^ bone marrow (scheme depicted in Figure 5E). In chimeras containing iCD4TR2 bone marrow, the frequency of mutant CD4^+^ T cells was increased significantly at 4 wk p.a. (unpublished data). Two weeks p.a. activation of CD4^+^ T cells and T_em_ cell proliferation were restricted to cells lacking TR2 (Figure 5F). The TR2-deficient T_n_ cell compartment was diminished while the mutant central memory compartment was unchanged (unpublished data). Analysis of control chimeras showed no differences between the CD45.1^+^ and CD45.2^+^ populations. These data thus suggest that TR2 regulates the homeostasis of mature T_em_ and T_n_ cells. While in models of constitutive TR2 ablation a large fraction of CD4^+^ T cells developed into IFN-γ–producing Th1 cells [11], we found only slightly increased IFN-γ production but no difference in T-bet levels after peripheral deletion of TR2 (Figure 5G). Production of Th2 cytokines was hardly detectable (Figure 5G) and the expression of the chemokine receptors CCR4, CCR5, CCR6, and CCR7 was unchanged (unpublished data).

Thus, hyperactivation, increased proliferation of T_em_ cells, and the reduction of the T_n_ cell compartment are cell-intrinsic consequences of TR2 ablation. The postthymic abrogation of TGF-β signalling does not lead to spontaneous acquisition of a Th1 phenotype.

### Increased TCR-Dependent Activation in Absence of TR2

**Figure 6:**
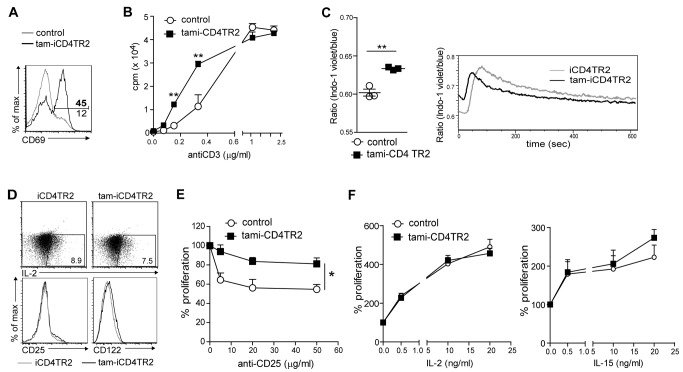
Deregulated proliferation control upon removal of TR2. (A) Flow cytometric analysis of CD69 expression by CD4^+^ splenic T cells isolated 2 wk p.a. These data are representative results of three independent experiments. (B) Analysis of sensitivity to activation through measurement of proliferation. Sorted CD4^+^ T cells were cultured for 72 h and stimulated with different anti-CD3 concentrations. Thymidine was added for the last 24 h of culture (mean ± SEM, 4 mice per group, analysed in two independent experiments). (C) Flow cytometric analysis of cytoplasmic calcium by ratiometric measurement of Indo-1–labelled cells from tam-iCD4TR2 and control mice. TCR crosslinking was performed after 15 s. On the left mean ratio of the baseline (representative data of two independent experiments). (D) Flow cytometric analysis of IL-2, CD25, and CD122 expression by splenic CD4^+^ T cells isolated from tam-iCD4TR2 and control mice. These data are representative results of two independent experiments. (E and F) Proliferation analysis of sorted CD4^+^ T cells cultured for 72 h with anti-CD3 (0.6 µg/ml) and anti-CD25 (PC61) (E) or indicated cytokines (F). Thymidine was added for the last 24 h of culture (mean ± SEM, 4 mice per group, analysed in two independent experiments).

Since the up-regulation of CD69 upon TR2 ablation (Figure 6A) supported the idea of a TCR-dependent effect [20] of TR2 signalling on proliferation rate of mature CD4^+^ T cells *in vivo*, we hypothesized that TR2 alters either the sensitivity to TCR ligands or the signalling through homeostatic cytokine receptors. First we assessed whether the absence of TR2 would result in increased sensitivity to TCR triggering. We stimulated CD4^+^ T cells in the presence of titrated amounts of anti-CD3 antibody and found an increased response to TCR stimulation through suboptimal anti-CD3 concentrations (Figure 6B), indicating an increased sensitivity to TCR stimulation after deletion of TR2. To exclude the possibility that this was the result of a larger fraction of experienced CD44^hi^ cells within the pool of stimulated TR2-deficient cells, we activated naïve and effector memory cells separately. Again the TR2-deficient cells, naive and experienced, presented with higher sensitivity to TCR stimulation (Figure S5A).

Indo-1–based ratiometric analysis revealed a small but significant increase in steady-state cytoplasmic calcium concentrations (Figure 6C). Upon stimulation the calcium influx was accelerated, suggesting an increased sensitivity of TR2-deficient T cells (Figure 6C). We did not find, however, differences in phosphorylation of CD3ζ, SLP76, lck, ZAP70, or ERK upon TCR stimulation (unpublished data). While no differences in CD25 expression or IL-2 production were found (Figure 6D), a slight increase of CD122 (IL-2Rβ) on CD4^+^ tam-iCD4TR2 T cells was detected 2 wk p.a. (Figure 6D). This could potentially have led to altered sensitivities to IL-15 or IL-2. However, the hyperproliferation of TR2-deficient T cells stimulated by suboptimal TCR activation could not be corrected by blockade of the IL-2R (Figure 6E and Figure S5B). Likewise, dose responses to IL-2 and IL-15 were equal in TR2^+^ and TR2^−^ CD4^+^ T cells (Figure 6F and Figure S5B). Furthermore, reactivity to IL-7 was unchanged in this *in vitro* assay (unpublished data). Thus, the hyperproliferative activity of TR2-deficient CD4^+^ T cells appears to be the result of increased TCR sensitivity but not of cytokine-mediated signals. To rule out that increased survival of TR2-deficient cells was contributing to the observed expansion, we removed the receptor from splenic CD4^+^ T cells *in vitro* and assessed survival. The result was an increased incidence of apoptosis by cells lacking TR2 as shown by Annexin V staining after 20 and 45 h (Figure S5C), thus excluding this possibility.

### Dysregulated Homeostasis of Regulatory T Cells After Ablation of TR2

**Figure 7:**
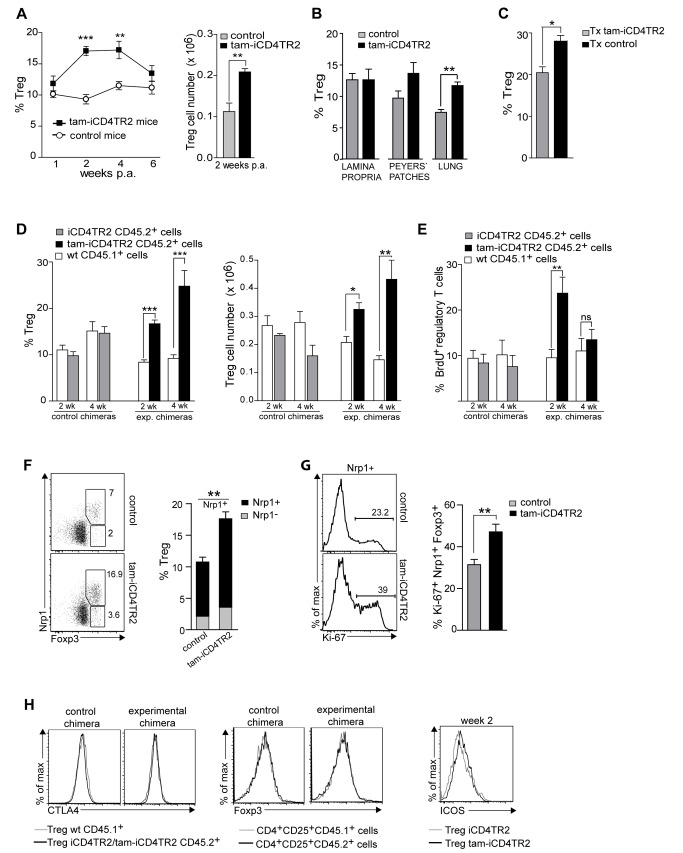
Increased proliferation of regulatory T cells upon removal of TR2. (A) The percentage of T_reg_ cells (left panel) and number of T_reg_ cells (right panel) in the spleen of tam-iCD4TR2 and control mice at indicated time points (mean ± SEM, 9 mice per group, analysed in two independent experiments). (B) The percentage of T_reg_ cells in the indicated organs of tam-iCD4TR2 and control mice (mean ± SEM, 5 mice per group, analysed in two independent experiments). (C) The percentage of T_reg_ cells in the spleens of thymectomised tam-iCD4TR2 and control mice 20 wk p.a. (mean ± SEM, 9 mice per group, analysed in two independent experiments). (D) The percentage of T_reg_ (left panel) and absolute number of Treg cells (right panel) within the LN CD4^+^ T cells of the indicated CD45.1^+^ or CD45.2^+^ bone marrow–derived cells (experiment described in Figure 5E, mean ± SEM, 10 mice per group, analysed in three independent experiments). (E) The percentage of BrdU^+^ T_reg_ cells isolated from LN (experiment described in Figure 5E, mean ± SEM, 10 mice per group, analysed in three independent experiments). (F) Flow cytometric analysis of Nrp-1 and Foxp3 expression by CD4^+^ T cells (left panel) and the percentage of Nrp-1^+^ and Nrp-1^−^ T_reg_ cells within the LN CD4^+^ T cells of tam-iCD4TR2 and control mice 2 wk p.a. (right panel) (mean ± SEM, 9 mice per group, analysed in two independent experiments). (G) Flow cytometric analysis of Ki-67 expression by Nrp-1^+^ T_reg_ cells (left panel) and the percentage of Ki-67^+^Nrp-1^+^ T_reg_ cells within the LN CD4^+^ T cells of tam-iCD4TR2 and control mice 2 wk p.a. (right panel) (mean ± SEM, 9 mice per group, analysed in two independent experiments). (H) Flow cytometric analysis of the expression of CTLA4 by T_reg_ and Foxp3 by CD4^+^CD25^+^ T cells isolated from spleen of mixed bone marrow chimeras. These data are representative results of three independent experiments (left panel). Flow cytometric analysis of the expression of ICOS by splenic T_reg_ cells from tam-iCD4TR2 and control mice at indicated time point p.a. Representative data of three independent experiments.

T_reg_ cell development and maintenance is generally thought to be critically dependent on TGF-β signals [21],[22] as thymic deletion of TGF-β signalling was shown to result in a strong reduction of T_reg_ cell number in the periphery [10],[11],[14]. When we examined T_reg_ cell homeostasis after peripheral ablation of TR2 from CD4^+^ T cells, we made the surprising observation of increased frequency and number of T_reg_ cells 2 and 4 wk p.a. as a result of hyperproliferation (Figure 7A and unpublished data). This increase in the T_reg_ population size was also observed in various LNs, the spleen, the lung, Peyer's patches, but not in the intestinal lamina propria (Figure 7B and unpublished data) and was stable in thymectomized animals (Figure 7C and unpublished data). Mixed bone marrow chimeras showed that similar to T_em_ cells, increased proliferation is an intrinsic property of the TGF-β unresponsive T_reg_ cells (Figure 7D,E and Figure S6A,B). The majority of the expanded splenic and LN Foxp3^+^ Treg cells in tam-iCD4TR2 mice expressed Nrp-1 and Helios (Figure 7F, Figure S6C, and unpublished data), which suggests a thymic origin [23]. Proliferation of TR-2–deficient Nrp-1^+^ regulatory T cells was increased compared to WT T_reg_ cells (Figure 7G). In thymic T_reg_ precursors lacking TGF-β signalling, a decrease in the levels of the key transcription factor FoxP3 [24]–[26] had been reported [27]. We did not observe such reduced Foxp3 levels in CD4^+^CD25^+^ cells in short- and long-term experiments (unpublished data) and in the mixed bone marrow chimeras (Figure 7H). Also, the expression of key surface molecules by TR2-deficient T_reg_ cells—namely CTLA-4, GITR (Tnfrsf18), [28],[29] and ICOS [30]—remained unchanged (Figure 7H) in contrast to our observations in the bone marrow chimera model presenting with a lethal autoinflammatory syndrome. We found, however, that as described for TR2-negative T_em_ also T_reg_ cells presented with an up-regulation of CD69 (Figure S6D). Therefore, to investigate TCR hyperresponsiveness, splenocytes isolated from tam-iCD4TR2 and control mice 2 wk p.a. were CFSE-labelled and stimulated with different concentrations of anti-CD3 antibody. Increased proliferation of TR2-negative T_reg_ cells was observed after 3 d of culture with suboptimal anti-CD3 concentrations (Figure S6E). Similarly, sorted T_reg_ cells showed hyperproliferation to anti-CD3–mediated stimulation when cultured alone or in presence of IL-2 (unpublished data). Taken together, TGF-β signalling suppresses overt T_reg_ cell proliferation but does not seem to be required for maintenance of the T_reg_ cell phenotype.

### TR2 Expression by T_reg_ Cells Is Not Required for Their Suppressive Capacity

**Figure 8:**
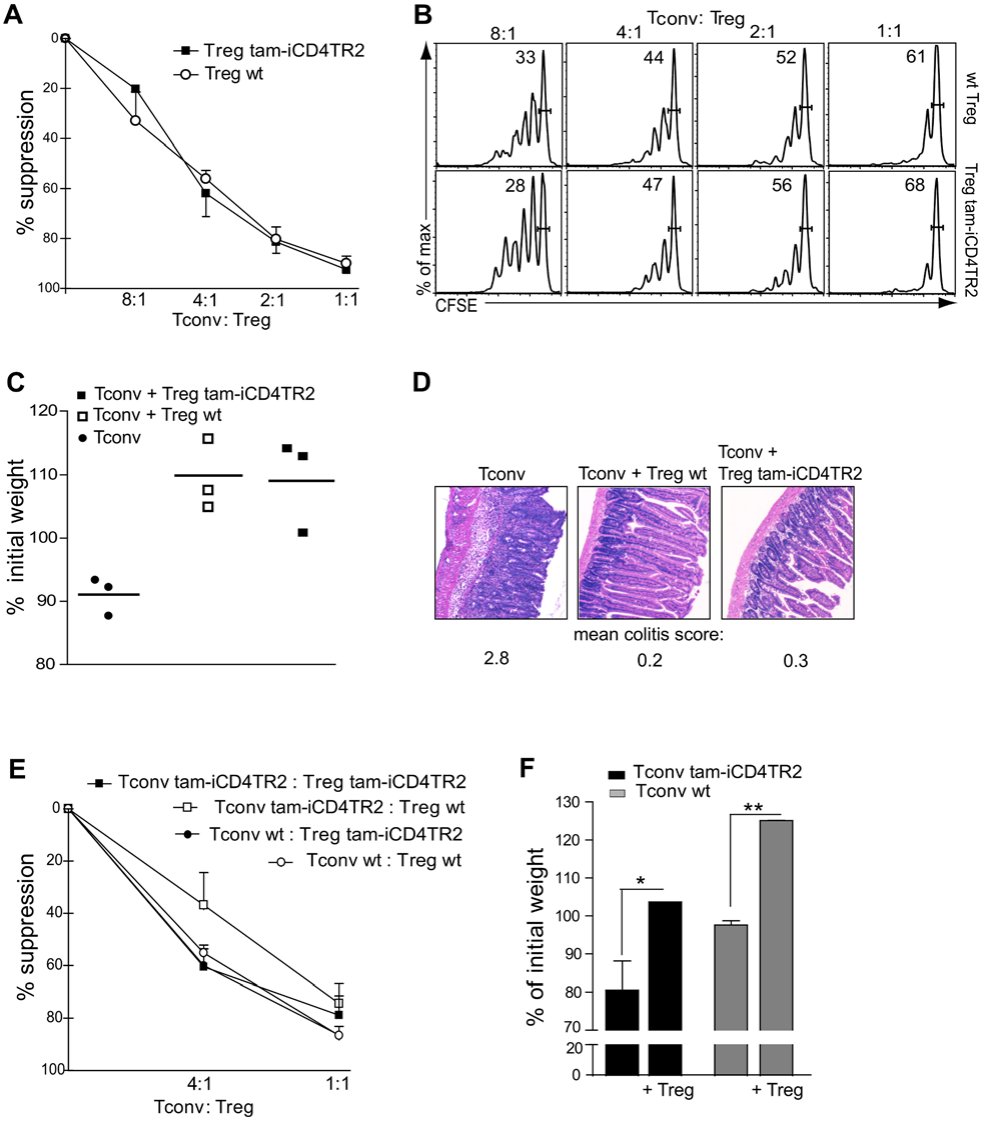
T_reg_ cells lacking TR2 are functional. (A) *In vitro* suppression assay: sorted conventional CD45.1^+^CD4^+^ T cells were stimulated with anti-CD3 (2 µg/ml) and cocultured with sorted WT T_reg_ cells or tam-iCD4TR2 T_reg_ cells (isolated 14 d p.a.) at various ratios. Thymidine was added for the last 24 h of culture. Analysis was performed after 96 h. Percent suppression as mean ± SD (analysed in two independent experiments). (B) *In vitro* suppression assay: sorted conventional CD45.1^+^CD4^+^ T cells were labelled with CFSE, stimulated with anti-CD3 (2 µg/ml), and cocultured with sorted wt T_reg_ cells or tam-iCD4TR2 T_reg_ cells (isolated 14 d p.a.) at various ratios. FACS analysis was performed after 96 h. These data are representative results of two independent experiments. (C and D) *In vivo* suppression assay: Development of colitis in Rag1^−/−^ mice after transfer of conventional CD4^+^ T cells alone or in combination with tam-iCD4TR2 (mice treated for 5 d, cells isolated 1 wk p.a.) T_reg_ cells or iCD4TR2 T_reg_ cells. Change in body weight after 8 wk posttransfer (mean, 3 mice per group, representative data of two independent experiments). (D) Representative micrographs of H&E-stained small intestine sections from *in vivo* suppression experiments isolated from Rag1^−/−^ mice 8 wk after transfer of the indicated cells. Scoring of colitis severity according to [60]. (E) Criss-cross *in vitro* suppression assay: sorted conventional tam-iCD4TR2 and WT T cells were cocultured with sorted tam-iCD4TR2 and wt T_reg_ cells at various ratios. Analysis was performed after 96 h (representative data of two independent experiments). (F) Development of colitis in Rag1^−/−^ mice after adoptive transfer of conventional tam-iCD4TR2 and wt T cells alone or in combination with tam-iCD4TR2 T_reg_ cells. Change in body weight after 8 wk posttransfer (mean ± SEM, 3 mice per group, representative data of two independent experiments).

Since peripheral TR2 ablation resulted in loss of cell cycle control and thus T_reg_ cell expansion, we tested whether these mutant T_reg_ cells retained functional characteristics identical to WT T_reg_ cells. In an *in vitro* suppression assay TR2-deficient T_reg_ cells and WT T_reg_ cells inhibited the proliferation of conventional T cells to a similar extent (Figure 8A,B). This observation was confirmed *in vivo* by the capacity of TR2-deficient T cells to suppress the development of colitis. TR2-deficient or control T_reg_ cells were co-transferred with CD4^+^ T_n_ cells into lymphopenic RAG1-deficient recipients. While transfer of T_n_ cells alone resulted in severe weight loss, indicative of colitis, the disease was similarly suppressed by co-transfer of T_reg_ cells from tam-iCD4TR2 or control animals (Figure 8C). Colon histopathology did not reveal any difference between mice that received TR2-deficient or WT T_reg_ cells (Figure 8D).

Given that T-cell–mediated autoimmunity upon thymic ablation of TGF-β signalling could not be suppressed by WT T_reg_ cells [11], we tested whether WT and TR2-deficient T_reg_ could suppress mutant responder T cells. In the *in vitro* as well as the *in vivo* suppression assay, TR2-deficient T cells remained susceptible to suppression by T_reg_ cells (WT and mutant) (Figure 8E,F). Taken together, TGFβ is not necessary for the functional capacity of T_regs_ to suppress immune responses.

## Discussion

The pleiotropic nature of the TGF-β family members has made it extremely challenging to unravel their function *in vivo*. All models of constitutive ablation of TGF-β signalling in αβT cells during thymic development have invariably revealed autoimmune phenotypes [5],[6],[9]–[11],[31]–[35]. In most of these models [10]–[12],[14] the T_reg_ cell population collapsed, resulting in almost complete loss of T_reg_-mediated peripheral suppression. These observations led to the dogma that TGF-β1 is required for establishment and maintenance of T cell tolerance. Yet because gene ablation in all these systems took place prior to or during thymic development, it could not be excluded that the observed immune dysregulation was a consequence of T cell development in the absence of TGF-β signals. In fact, while Doisne and colleagues showed that NKT cells depend critically on TGF-β for their development [13], Ouyang and colleagues reported that even conventional T cell development is modified by TGF- β signalling [12]. Therefore, to study the importance of TGF-β for peripheral CD4^+^ T lymphocytes, we circumvented such thymus-related abnormalities through induced deletion of TR2 in mature T helper cells only by use of a novel CD4-CreER^t2^ system. It allowed induced recombination of the TR2 target allele within up to 95% of postthymic CD4^+^ T cells. Recombination during thymic development remained small after short-term tamoxifen application, and we did not find a contribution of TR2-deficient CD8^+^ T cells to the peripheral T cell pool in this setup. Even long-term treatment with tamoxifen resulted in recombination in only a small fraction of CD8^+^ T cells. Only in one experimental setup, when thymic development was initiated in the presence of tamoxifen, did we observe a higher recombination frequency in CD8^+^ T cells. Therefore, our CD4-CreER^t2^ system proved to be useful for the study of TR2 function in peripheral CD4^+^ T cells. The reason for the small recombination rate in DP thymocytes may lie in the fact that the cells reside only shortly at this stage. Also, during this stage expression of CreER^t2^ is initiated and protein has to first accumulate. Therefore only a small number of DP cells is receptive and exposed to tamoxifen at the same time. Further, only a minor fraction of T cells is generated newly during the 5-d treatment setup and can thus contribute to the peripheral pool. Another factor influencing efficiency of Cre-mediated recombination is the accessibility of the target allele. Thus the extent of recombination in the thymus may depend on the target used as well as duration and route of tamoxifen application.

Taken together by using the tam-iCD4TR2 mouse for the analysis of TGF-β's role for CD4^+^ T cells, we report (i) the absence of clinical or immunological manifestations of autoimmunity, even when removal of TR2 from CD4^+^ T cells was maintained for months; (ii) the development of a lethal autoimmune syndrome only when TR2 ablation is induced during thymic development; (iii) an increase in T_em_ and T_reg_ proliferation as a result of increased TCR signal sensitivity in the absence of TR2; and (iv) that TGF-β signalling is not required for the maintenance of the suppressive capacity of mature T_reg_ cells *in vitro* and *in vivo*. We therefore propose that the widely held notion of TGF-β signalling being a major contributor to peripheral T cell tolerance and T_reg_ cell maintenance results from observations of developmental aberrations due to the use of complete gene ablation during thymic development under lymphopenic conditions.

Since the autoimmune syndrome in the most drastic cases of TGF-β signal manipulation results in lethality at a very young age, we hypothesized that neonatal lymphopenia was a contributing factor. To distinguish between effects through mere lymphopenia and aberration in thymic development, we performed TR2 ablation in three different lymphopenic setups, but all of them failed to drive systemic autoinflammation. One condition, adoptive transfer of TR2-deficient T cells into a lymphopenic host, resulted solely in colitis, similar to a recently published study of dLck-Cre–driven TR2 ablation [15]. We could show that the difference between development of colitis (CD4-CreER^t2^/TR2^f/f^, dLck-Cre/TR2^f/f^) and a lethal systemic autoimmune syndrome (our thymic deletion model, CD4-Cre/TR2^f/f^, Lck-Cre/TR1^f/f^) lies in whether TGF-β signalling is disrupted later than or at the DP thymic stage, respectively. By use of the acute lymphopenia models of sublethal irradiation and anti-CD4 depleting antibody treatment, we focused on systems which were shown to drive T cell proliferation by increased availability of IL-7, a setting which amplifies signals from weak TCR/self-pMHC contacts [36],[37]. We again observed hyperproliferation and basically complete conversion to a (post)activated phenotype (unpublished data), yet neither organ-specific nor systemic autoimmunity developed. A difference in recombination frequencies could have been another cause of the difference in development of autoimmunity between the inducible and cell-type–specific systems, but we and others [10],[11] provided evidence to rule out this possibility. The decrease of T_reg_ cell numbers or of their suppressive activity in the constitutive models are also an unlikely cause for development of autoimmunity since activated TR2-deficient T cells in these models were shown to be resistant to control by even WT T_reg_ cells [10],[11],[27]. Taken together, minor differences in gene deletion efficiency as well as the presence/absence of T_reg_ cells cannot account for the divergent autoimmune phenotypes of the constitutive and our inducible model. Also, impaired thymic negative selection was ruled out by a study that found actually a slight increase of negative selection in absence of TR2 [12]. Another possibility was that TR2 was not ablated in the T cell subset required for development of autoimmunity, but several studies indicated that both CD4^+^ and CD8^+^ T cells are responsible for the autoimmune phenotype in absence of TGF-β signalling [10],[38],[39]. We excluded this possibility by depletion of CD8^+^ T cells in the setup. This resulted also in lethal autoimmunity, albeit slightly delayed, and did not prevent weight loss and infiltration into the lung and liver. Further, a recent study showed that TR2-deficient CD4^+^ T cells alone can induce autoimmune colitis under lymphopenic conditions [15], an observation that we confirmed in our model. One report placed the pathogenic activity of TR2-deficient T cells in unconventional NK T cells [10], but we show that NK1.1 expression is not a mandatory characteristic of such autoreactive T cells. Taken together, the only common denominator for development of severe lethal autoimmunity upon removal of TGF-β signalling in T cells is that recombination must occur in developing thymocytes in a severely lymphopenic animal. While a recent study reported that also the abrogation of TGF-β signalling in CD11c^+^ cells leads to the development of autoimmune disease [40], this seems unlikely in our system since recombination in CD4^+^ CD11c^+^ cells was very rare.

When we used our model to analyze the role of TGF-β signalling for survival and homeostasis of peripheral CD4^+^ T cells, it revealed a modest increase in size of the T_em_ and T_reg_ compartments with both populations being activated (CD69^+^) and cycling (BrdU^+^). For T_em_ cells such an effect has also been reported when TGF-β signalling was abrogated by CD4-Cre or through a CD4 promoter-driven dominant-negative TR2 transgene, but the extent was far stronger than in our model [5],[10],[11]. In all such models the hyperproliferation and activation in CD8^+^ T cells was considerably larger than in CD4^+^ T cell compartment [5],[10],[11].

As reported before [8],[10],[11] this hyperproliferation of TGF-β unresponsive T cells depends on specific peptide-MHC recognition. The introduction of a TCR transgene, whose antigen is not recognized in the periphery [41], into our model resulted in amelioration of the proliferative phenotype (unpublished data). This is supported by data from dLck-Cre–mediated TR2 ablation, acting in late thymic development. In this model combination with OT-I TCR transgene showed increased TCR sensitivity when peptides of different affinities were used [15]. Similarly, we also found increased sensitivity to TCR stimulation *in vitro* in the absence of TR2, which may be a result of increased steady-state intracytoplasmic calcium concentrations and faster calcium response. Taken together, in all models of abrogation of TGF-β signalling in T cells the threshold of productive TCR signalling is decreased.

Even though we and Zhang and Bevan showed that increased sensitivity of TCR signalling is a major factor in the increased proliferative phenotype of TR2-deficient T cells, also direct control of cell cycle by TGF-β signalling has been reported [42]–[45]. Such cell-cycle control appears to be connected to the establishment of T cell tolerance (anergy) in the DO11.10 model, thus again highlighting the importance of TGF-β in maintaining T cell homeostasis [45].

As an additional cause of hyperproliferation and activation of TGF-β unresponsive T cells, dysregulated sensitivity to γc cytokines has been reported. The homeostasis especially of CD8^+^ memory T cells was shown to be under IL-15 control, possibly due to higher expression of CD122 in comparison to CD4^+^ memory T cells (for rev: [37],[46]). In agreement with this, a CD2 promoter-driven dominant-negative TR2 transgene was found to present with a IL-15–dependent expansion of memory-like CD8^+^ T cells [8]. The T-bet–mediated up-regulation of CD122 in CD4-Cre TR2 model, however, was observed in both CD4^+^ and CD8^+^ T cells and was considered to be central for maintenance and expansion of TGF-β–unresponsive memory T cells [11]. In our inducible model we found neither evidence of increased IL-2 production by activated CD4^+^ T cells nor increased expression of CD25 (IL-2 Rα). We observed, however, a slight increase in CD122 expression (IL2Rβ chain) upon induced ablation of TR2 in CD4^+^ T cells. Yet this did not lead to increased sensitivity to common gamma chain cytokines, making them an unlikely cause of the hyperproliferation seen in T_em_ and T_reg_ cells. Thus, the impairment of TGF-β signalling in peripheral T cells leads to hyperproliferation independent of cytokine signalling, while constitutive ablation during thymic development results in dysregulated γc cytokine production/signalling and massive autoimmunity.

We therefore conclude that TGF-β controls postthymic homeostasis of both naïve and memory CD4^+^ T cells. Yet the extent of hyperproliferation in induced TR2-deficient T cells is significantly lower than in constitutive TR2 mutants.

Previously, the almost complete absence of peripheral T_reg_ cells in CD4-Cre/TR2^f/f^ animals was taken as evidence for a prominent role of TGF-β in T_reg_ cell maintenance [11]. In this model T_reg_ cells and precursors also showed a hyperproliferative phenotype [11], but in the periphery T_reg_ cells died prematurely [12]. Also, the drastic expansion of thymic T_reg_ cells in TR1-deficient thymocytes [14] resulted in their failure to survive in the periphery. In contrast to these observations, upon peripheral TR2 ablation T_reg_ cells show increased proliferation without associated collapse through massive apoptosis. This expansion of TR2-negative T_reg_ cells in peripheral lymphoid organ in our model was restricted to Nrp-1^+^ Foxp3^+^ T_reg_ cells, most likely cells of thymic origin [23]. A specific increase of nT_reg_ cells upon TR2 ablation is also supported by the increase in number of Helios^+^ T_reg_ cells [47]. Conversely and similar to our observation, TGF-β1 gene ablation from activated peripheral T cells resulted in an expansion of T_reg_ cells [48], possibly a consequence of decreased local TGF-β availability. Expansion of T_reg_ cells could have been the result of IL-2 production by the increased number of activated T cells in tam-iCD4TR2 animals, but our data excluded this possibility. Also, only mutant T_reg_ cells showed increased cycling in mixed bone marrow chimeras, thus ruling out any effects in trans. The up-regulation of CD69 by T_reg_ cells and *in vitro* stimulation with suboptimal anti-CD3 concentrations suggest increased TCR sensitivity, similar to conventional T cells.

We are to our knowledge the first to study T_reg_ cell physiology after induced peripheral abrogation of TGF-β signalling *in vivo*. Our finding of the TGF-β independence of the mature, postthymic T_reg_ phenotype and function does agree with epigenetic imprinting taking place at the Foxp3 locus and also many other loci relevant for T_reg_ cell physiology [49]. Upon induced TR2 ablation we observed neither reduction of Foxp3, CTLA-4, and GITR levels nor any decrease in the suppressive capacity of TR2-deficient T_reg_ cells. Tone and colleagues [50] showed that TGF-β–induced Smad-mediated activation of the Foxp3 locus through interaction with a conserved Smad-NFAT response element (CNS1) in thymocytes is essential for nT_reg_ cell generation. Once the T_reg_ phenotype is established, expression of FoxP3 in nT_reg_ cells is largely independent of the promoter region CNS1 [51],[52], which is supported by our observation of unchanged FoxP3 levels. Further, Miyao and colleagues recently showed that T_reg_ cells represent a stable lineage, which robustly maintains its committed state once the Treg cell-specific demethylation region (TSDR) has been demethylated [53].

TGF-β receptor signalling is, however, required for the induction of Foxp3 among peripheral naïve CD4^+^ T cells (iT_regs_) [54],[55]. In agreement with this, we found that in the lamina propria, where iT_reg_ cells contribute substantially to the T_reg_ pool, no T_reg_ expansion can be observed upon induced TR2 ablation.

Finally, we could show that the proliferation of TR2-deficient effector cells can be inhibited by TR2-deficient T_reg_ cells, a question that has been raised recently [56]. Thus, we propose that ablation of TGF-β signalling during thymic development leads to intrathymic hyperproliferation of T_reg_ cells, which then cannot survive in the periphery. Conversely, when TR2 is removed from already established peripheral T_reg_ cells, these cells keep their T_reg_ cell characteristics and undergo increased proliferation.

Taken together, our study suggests that several misconceptions about TGF-β function for mature T cells are the result of gene ablation during T cell development. By restricting the genetic defect (TR2-deficiency) to mature CD4^+^ T cells, we show that TGF-β signalling is not essential for the suppression of autoimmunity. Furthermore, it is not obligatory for the maintenance of a functional T_reg_ cell pool. Instead it is required for the homeostasis of T_reg_ and T_em_ cells by curbing TCR signalling and therefore overt proliferative activity. In contrast, ablation of TGF-β signalling during thymic development as well as during lymphopenia may predispose for development of autoimmunity. Thus, TGF-β1 remains a cytokine with critical function in the regulation of T cells, yet its role in peripheral tolerance has been overestimated.

## Materials and Methods

### Generation of the CD4-CreER^t2^ Allele

Targeting of the CreER^T2^ fusion gene to the CD4 locus was achieved by replacing part of exon 2 including the start codon with the targeting vector *pBluescript CD4-Cre19ER^T2^* by homologous recombination in the C57BL/6 derived ES cell line Bruce4. Colonies were analyzed for homologous recombination events by Southern blot analysis of Hind*III* digested genomic ES cell DNA. Two positive clones were used for blastocyst injection. Probes were amplified with: *3′PROBE:* AAC TGC ACC GTG ACC CTG GAC CAG AAA AAG AA and GTA GGA GTG AAG GTC AGA GAC CAG GAC AAT AG; and *5′PROBE:* CTT CAA ATA ATT AAC AAA ACA ACA AAA CCC TT and AAA AAC CAA AAC CAA CCC AAA CAA AAA ACA T.

### Animal Maintenance and Experiments

*Tgfbr2^fl/fl^* [57] were kindly provided by U. Malipiero, and ROSA-EYFP mice [58] were kindly provided by A. Diefenbach, C57BL/6J (B6) mice were purchased from Charles River and congenic C57BL/6-CD45.1 bred in-house. CD4-CreER^t2^, CD4-Cre, RAGE, TGFβRII^fl/fl^, B6, B6-CD45.1, Rag1^−/−^, and ROSA-EYFP mice were maintained in barrier and specific pathogen-free facilities at the University of Zurich and Technical University of Munich and handled in accordance with approved protocols under permits of the cantonal veterinary office. Organs and sera of fas-deficient animals were provided by Bojan Polic (Rijeka, Croatia).

For genotyping of CD4-CreER^t2^ mice, the following primers were used: GCC AGC TCA TTC CTC CCA CTC, CAT GGG ACT TTG GGC TTC TAG G and CCC AAC CAA CAA GAG AGC TCA AG, amplifying wt (440 bp) and transgene (720 bp). Genotyping of TR2 was performed according to [57].

If not stated otherwise all mice used for the experiment were at the age of 6 to 10 wk.

For tamoxifen (Sigma) application, tamoxifen was dissolved in 100% ethanol to 1 g/ml, vortexed, and mixed with olive oil to a final concentration of 100 mg/ml. The suspension was incubated at 56°C for 15 min and sonicated for 20 min. iCD4TR2 mice were force-fed with 5 mg tamoxifen per day for 5 consecutive days. Day 7 after start of application is denoted 1 wk p.a. For long-term treatment, mice were fed with tamoxifen citrate (Harlan) for 8 or 12 wk ad libitum.

For induction of lymphopenia, mice were irradiated with 550 rad or injected i.p. once with 10 µg GK1.5 anti-CD4.

For depletion of CD8^+^ T cells, mice were injected at day 15 and 17 post–bone marrow transplantation with 250 µg of anti-CD8 antibody (YTS 169.4 clone, BioXcell) followed by 100 µg every week. Controls received the same amount of isotype control antibody LTF.2 (rat IgG2b, BioXcell).

Bone marrow chimeras were generated by i.v. transfer of T cell–depleted bone marrow cells (1×10^7^) into lethally irradiated (1,100 rad) Rag1^−/−^ mice. Thymectomies were performed under Ketamine/Xylazine anaesthesia according to published procedures [59].

For *in vivo* proliferation analysis BrdU (80 mg/100 ml) was added to drinking water and changed every second day for 7 d. Intracellular staining with anti-BrdU antibody (eBioscience) and Foxp3 Staining Buffer Set (eBioscience) followed by DNAse (Invitrogen) treatment for 1 h in 37°C was performed. Samples were analyzed by using FACS Canto II.

For the *in vivo* suppression assay, Rag1^−/−^ mice were injected intraperitonelly with 4×10^5^ conventional T cells (CD45.1^+^) alone or in combination with 2×10^5^ regulatory T cells (CD45.2^+^). Mice were weighed and assessed for clinical sings of colitis weekly and were killed 9 wk after transfer. Colons were fixed in 4% formalin, paraffin-cut, and stained with hematoxylin and eosin according to standard procedures.

For adoptive transfer experiments, Rag^−/−^ mice were injected intraperitonelly with 2×10^6^ purified T cells (T cell isolation kit, Milteny). Mice were weighed, assessed for clinical symptoms of colitis weekly, and killed 7 wk after transfer.

### Flow Cytometric Analyses

For flow cytometry the following fluorochrome conjugated antibodies were used: anti-CD3, anti-CD4 (L3T4), anti-CD8α (53-6.7), anti-CD8β (YTS 156.7.7), anti-CD19, anti-CD25, anti-CD44, anti-CD45, anti-CD45.1, anti-CD45.2, anti-CD45RB, anti-CD49b, anti-CD62L, anti-CD69, anti-CD122, anti-CD154, anti-GITR, anti-NK1.1, anti-CCR4, anti-CCR5, anti-CCR6, anti-CCR7, anti-IL2, anti-Ki-67, anti-INF-γ, anti-IL-4 all from BD Biosciences, anti-TGF-βRII from R&D, anti-Ly6A/E, anti-CD90.2 from BioLegend anti-Foxp3, and anti-BrdU from eBiosciences from Cell Signalling Technology. Staining for Foxp3, BrdU, and Ki67 was performed using Foxp3 Staining Buffer Set (eBiosciences) according to the manufacturer's protocol. For intracellular cytokine staining, cells were stimulated for 4 to 6 h with 50 ng/ml PMA and 500 ng/ml Ionomycin and 1 µl/ml GolgiPlug (BD Bioscience). Samples were acquired using FACS CantoII (BD Biosciences) and analyzed with FlowJo software (Treestar).

To enrich CD4^+^ T cells, population magnetic sorting was performed (CD4 T cell isolation kit, Milteney Biotech) according to the manufacturer's protocol.

T_reg_ cells and T_conv_ cells were sorted on a FACS Aria (BD Biosciences) on the basis of being CD4^+^CD45RB^lo^CD25^+^ and CD4^+^CD45RB^hi^CD25^−^, respectively. Naïve CD4 T cells were sorted on the basis of being CD4^+^CD44^lo^CD62l^hi^, effector memory/experienced CD4^+^CD44^hi^CD62l^lo^, and central memory CD4^+^CD44^hi^CD62l^hi^. For proliferation assays naïve and memory CD4^+^CD25^−^ T cells were sorted.

### Measurement of TCR Signalling

For calcium analysis, lymph node cells were labelled with Indo-1 AM (1.5 µM, Invitrogen) for 45 min in 37°C, washed with 2% FCS RPMI medium, and subsequently labelled with anti-CD8, anti-CD19, anti-NK1.1, and anti-CD11b antibodies for 20 min in 4°C. Cells were resuspendet in 2% FCS RPMI, surface-labelled with anti-CD3 (10 µg/ml final, eBioscience), and anti-CD28 antibody (10 µg/ml final, eBioscience) for 10 min on ice followed by 10 min at 37°C. After addition of propidium iodide, a 15 s baseline was acquired followed by TCR activation through crosslinking with anti-hamster antibody (24 µg/ml final, Jackson ImmunoResearch Laboratories). Samples were acquired using LSR Fortessa (BD Biosciences) and analysed by FlowJo software (Tristar).

### Histological Tissue Analyses

Mice were euthanized with CO_2_, perfused first with PBS, and then 4% paraformaldehyde in PBS. For histological analysis, kidney, liver, heart, colon, and small intestine were fixed in 4% paraformaldehyde in PBS, paraffin-embeded, cut into 30 µm thick sections, and stained with hematoxylin-eosin and anti-CD3 antibody according to standard procedures.

### *In Vitro* Proliferation Assay

Cells from the spleen or lymph nodes were cultured in RPMI 1640 (Invitrogen) medium supplemented with 10% FCS, 1% penicillin-streptomycin, 0.5% 2-mercaptoethanol, stimulated for 72 h with different concentrations of anti-CD3 (2C11, BioXcell) alone or together with 5 µg/ml anti-CD28 (N37, BioXCell). For CFSE labelling, cells were first stained for 20 min in the dark at room temperature with CFSE (carboxyfluorescein diacetate succinimidyl diester, 5 µM) and washed in PBS. For thymidine incorporation assays, 1 µCi of thymidin per well was added for the last 24 h of cultures. For blocking of IL-2 signalling, the anti-CD25 antibody (PC61, eBioscience) at different concentrations was added to the culture together with anti-CD3 antibody. For assessment of the role of common γ-chain cytokine on proliferation, IL-2 and IL-15 (Peprotech) were added to the culture at different concentrations together with anti-CD3.

### *In Vitro* Suppression Assay

The 5×10^4^ sorted conventional T cells and T_reg_ cells (in ratios according to figure legend) were cultured for 96 h in round-bottomed plates along with anti-CD3 antibody (2C11, 2 µg/ml) and irradiated splenocytes in RPMI 1640 medium supplemented with 10% FCS, 1% penicillin-streptomycin, and 0.5% 2-mercaptoethanol. T cell proliferation was determined by thymidine incorporation and CFSE labelling of T_conv_ cells as described above.

### *In Vitro* Apoptosis Assays

Lymphocytes from LN and spleens were stained for CD4, CD44, CD62L, CD25, CD45RB, and sorted by using FACS Aria. The purity for each population was above 95%. Cells were cultured in AIM-V medium (Invitrogen) supplemented with 0.05% 2-mercaptoethanol (Sigma) without stimulation. After 20 and 40 h, staining for Annexin V (BD Bioscience) and Topro3 or propidium iodide (1 µg/ml) was performed.

### Quantitative Real-Time PCR Analysis

Different subsets of CD4 T cells were isolated by FACS sorting and used for mRNA extraction (RNeasy mini kit, Qiagen). mRNA was transcribed with M-MLV reverse transcriptase (Invitrogen). Quantitative RT-PCR was performed with MyIQ cycler (Biorad) using SyberGreen (Invitrogen). The following primers were used: TR2: AAC GAC TTG ACC TGT TGC CTG T and CTT CCG GGG CCA TGT ATC TT; and T-bet: CAA CAA CCC CTT TGC CAA AG and TCC CCC AAG CAG TTG ACA GT. The expression of the genes was standardized to the relative quantity of RNA polymerase 2 detected by the primers CTG GTC CTT CGA ATC CGC ATC and GCT CGA TAC CCT GCA GGG TCA and normalized to the value of the respective control condition.

### Enzyme-Linked Immunosorbant Analysis (ELISA)

For detection of anti-dsDNA antibodies from the serum, ELISA plates (BD Falcon) were pretreated with 0.1% poly-L-lysine (Sigma) for 2 h, coated with 100 µg/ml DNA (Sigma) overnight, and blocked with 2% BSA for 2 h. After washing with PBS, anti-mouse IgG γ chain HPR-conjugated antibody (Sigma) was applied for 45 min and developed with stabilised chromogen (Invitrogen).

### Immunoblotting

To analyze Smad2 phosphorylation MACS sorted CD4^+^ T cells from tam-iCD4TR2 and control mice were stimulated with 20 ng/ml TGF-β1 (Peprotech). Western blot analysis was performed after cell lysate separation in 10% SDS-PAGE gels with anti-pSmad2 (Cell Signaling Technology) and a donkey anti-rabbit Ig-G HRP secondary antibody.

### Immunofluorescent Staining

NIH3T3 cells were cultured on poly-d-lysine (Sigma) precoated glass slides. Cells were fixed with methanol (Fluka) for 10 min and permeabilized with 1% Triton X-100 (Sigma) for 20 min. After blocking with 10% normal goat serum (Dako), cells were incubated overnight with serum diluted 1∶200 in 1% goat serum and 0.2% Triton X-100 (Sigma). Cells were incubated with goat anti-mouse IgG antibody AlexaFluor 546 conjugated (Invitrogen) for 45 min, and the nuclei were counterstained with Hoechst 33342 (Invitrogen). Analysis was performed on Olympus IX81 microscope with CellM software.

### Statistics

The *p* values were calculated with Student's *t* test using Prism software. The *p* values of less than 0.05 were considered significant. Where no *p* value is indicated through stars, no statistically significant difference was found.

## Supporting Information

Figure S1 **Targeting strategy of the CD4-CreER^t2^ mouse.** (A) Schematic map of the targeting strategy for the CD4 Locus. The Cre-ER^T2^ open reading frame and an FRT-flanked neomycin resistance gene were inserted into exon 2 of the CD4 locus of murine ES cells. HindIII restriction sites used for Southern blot analysis of the targeted ES cell are indicated. (B) Southern blot screen of the targeted ES cells after digestion with HindIII and hybridization with the 5′ external probe and 3′ external probes. Homologous recombination is indicated by newly appearing 2.9 kb and 8.8 kb bands for the targeted allele in addition to the 10.6 kb band for the WT allele.
(TIF)

Figure S2 **Deletion efficiency of TR2 in the thymus and spleen.** (A) Flow cytometric analysis of TR2 expression by splenic CD4^+^ and CD8^+^ T cells (left panel). Flow cytometric analysis of TR2 expression by splenic effector memory, naïve and CD25^hi^ CD4^+^ T cells (right panel). These are representative data of three independent experiments. (B) Quantitative RT-PCR of TR2 mRNA in FACS-sorted thymocytes subsets 1 and 2 wk p.a. These data are representative results of two independent experiments.
(TIF)

Figure S3 **Schemes of experimental setups and FACS analysis of TR2 deletion in respective experimental setups.** (A) The scheme of the experiment described in Figure 3A–G. (B) The scheme of the experiment described in Figure 3H. Flow cytometric analysis of TR2 expression by CD4^+^ and CD8^+^ T cells from peripheral blood after long-term tamoxifen citrate treatment. (C) The scheme of the experiment described in Figure 3J–K. Flow cytometric analysis of CD4^+^ and CD8^+^ T cell frequencies in the spleen of chimeric mice at day 55 following anti-CD8α (YTS 169.4) or isotype control treatment.
(TIF)

Figure S4 **Schemes of experimental setups and FACS analysis of TR2 deletion in lymphopenic environment.** (A) Scheme of the experiment described in Figure 4A and flow cytometric analysis of TR2 expression by CD4^+^ and CD8^+^ T cells from peripheral blood after long-term tamoxifen citrate treatment (day 90). (B) Scheme of the experiment described in Figure 4C. (C) The percentage and number of CD4^+^ T cells (left panel) and CD8^+^ T cells (right panel) in the mesenteric lymph nodes of Rag^−/−^ mice 7 wk after adoptive transfer of tam-iCDTR2 and control T cells (mean ± SEM, 5 mice per group, analysed in two independent experiments).
(TIF)

Figure S5 **Proliferation of TR2-deficient CD4^+^ T cells.** (A) Sorted effector memory and naïve CD4^+^CD25^−^ T cells were cultured for 72 h and stimulated with indicated concentrations of anti-CD3 antibody. Thymidine was added for the last 24 h of culture (mean ± SEM, 4 mice per group, analysed in two independent experiments). (B) Proliferation analysis of sorted CD4^+^ T cells cultured for 72 h with anti-CD3 (0.6 µg/ml) and anti-CD25 (PC61) or with indicated cytokines. Thymidine was added for the last 24 h of culture. (C) *In vitro* analysis of apoptosis induction. Tam-iCD4TR2 and control cells were cultured in AIM-V medium with or without tamoxifen. The ratio between AnnexinV positive CD4^+^ T cells that were tamoxifen-treated versus untreated is indicated (mean, 3 mice per group). These data are representative of three independent experiments.
(TIF)

Figure S6 **Foxp3 and Helios expression by TR2-deficient regulatory T cells.** (A) Flow cytometric analysis of the expression of Foxp3 and CD25 by CD4^+^ T cells isolated from LN at 2 wk p.a. (B) Flow cytometric analysis of the BrdU positive T_reg_ cells in experimental and control chimeras 2 wk p.a. (C) Flow cytometric analysis of the expression of Helios and Foxp3 by CD4^+^ T cells isolated from LN at 2 wk p.a. These are representative results of two independent experiments. (D) Flow cytometric analysis of CD69 expression by splenic T_reg_ cells isolated from tam-iCD4TR2 and control mice from indicated experimental setups. (E) Proliferation analysis of T_reg_ cells *in vitro*. Splenocytes isolated from tami-CD4TR2 and control mice 2 wk p.a. were labelled with CFSE and cultured for 72 h with indicated concentrations of anti-CD3 antibody. Each row represents proliferation of T_reg_ cells from a different mouse. These are representative data of two independent experiments.
(TIF)

## Abbreviations

DN: double negative thymocytes
DP: double positive thymocytes
iCD4TR2: CD4-CreER^t2^/TGFbRII^f/f^
LN: lymph node
LP: lamina propria
mLN: mesenteric lymph node
p.a.: postapplication
tam-iCD4TR2: tamoxifen-treated CD4-CreER^t2^/TGFbRII^f/f^
T_em_: effector memory/experienced T cells
TGF-β: transforming growth factor β
T_n_: naïve T cells
T_reg_: regulatory T cells
TR1: TGF-βRI, transforming growth factor β receptor I
TR2: TGF-βRII, transforming growth β factor receptor II
Tx: thymectomised mouse
