# Supplementary figures and images for: TGF-β Signalling Is Required for CD4+ T Cell Homeostasis But Dispensable for Regulatory T Cell Function
Source: PLoS Biol. 2013 Oct 8;11(10):e1001674. doi: 10.1371/journal.pbio.1001674 (PMC3792861; doi:10.1371/journal.pbio.1001674)

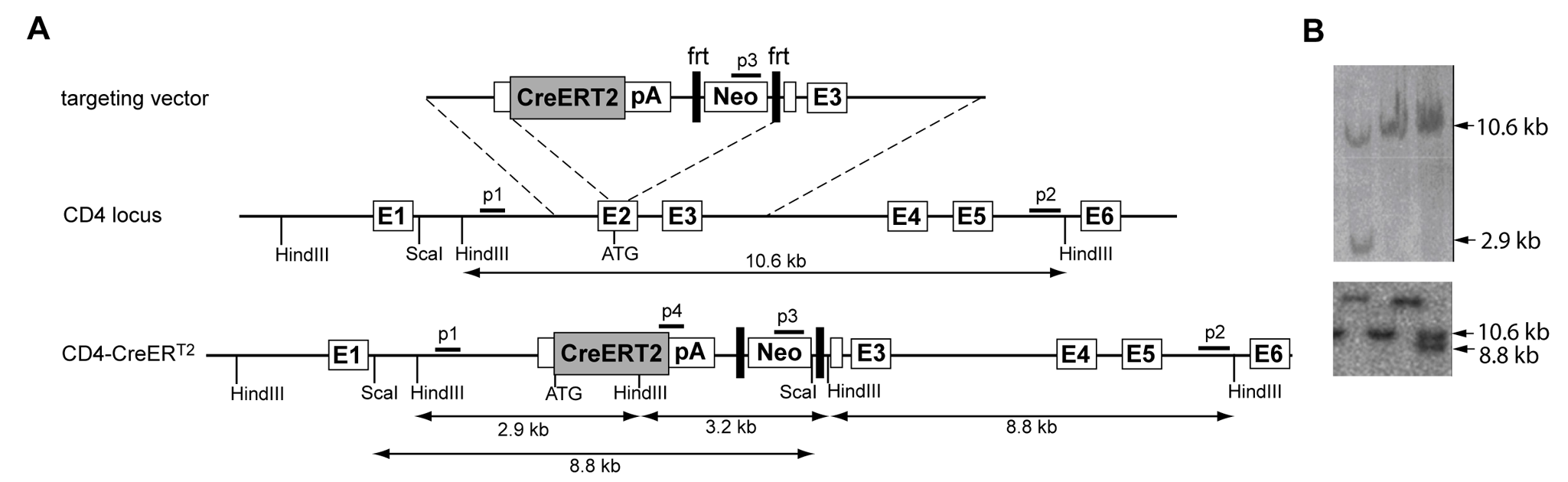

Supplement: Figure S1 — Targeting strategy of the CD4-CreERt2 mouse. (A) Schematic map of the targeting strategy for the CD4 Locus. The Cre-ERT2 open reading frame and an FRT-flanked neomycin resistance gene were inserted into exon 2 of the CD4 locus of murine ES cells. HindIII restriction sites used for Southern blot analysis of the targeted ES cell are indicated. (B) Southern blot screen of the targeted ES cells after digestion with HindIII and hybridization with the 5′ external probe and 3′ external probes. Homologous recombination is indicated by newly appearing 2.9 kb and 8.8 kb bands for the targeted allele in addition to the 10.6 kb band for the WT allele. (TIF) [file pbio.1001674.s001.tif]

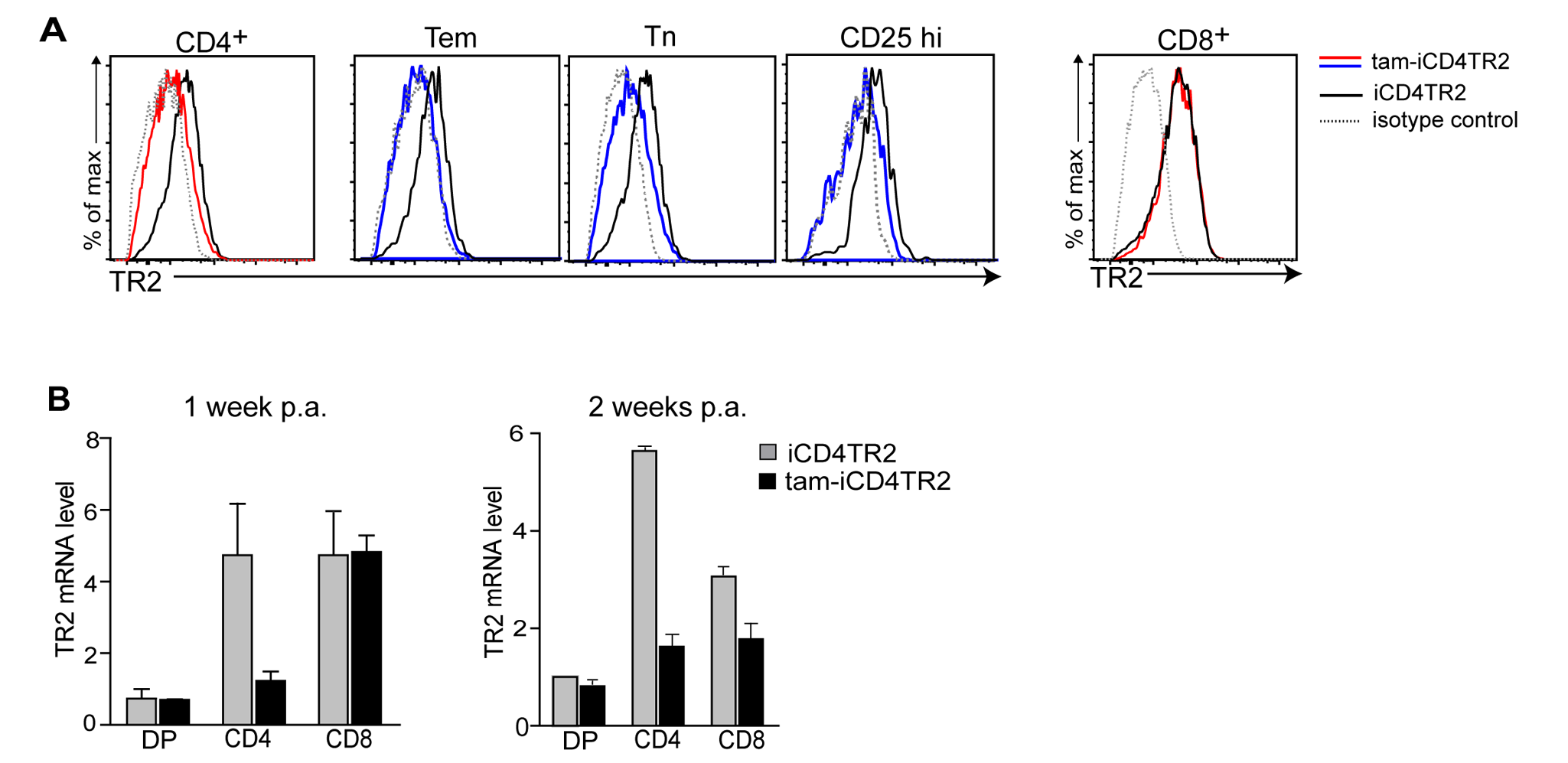

Supplement: Figure S2 — Deletion efficiency of TR2 in the thymus and spleen. (A) Flow cytometric analysis of TR2 expression by splenic CD4+ and CD8+ T cells (left panel). Flow cytometric analysis of TR2 expression by splenic effector memory, naïve and CD25hi CD4+ T cells (right panel). These are representative data of three independent experiments. (B) Quantitative RT-PCR of TR2 mRNA in FACS-sorted thymocytes subsets 1 and 2 wk p.a. These data are representative results of two independent experiments. (TIF) [file pbio.1001674.s002.tif]

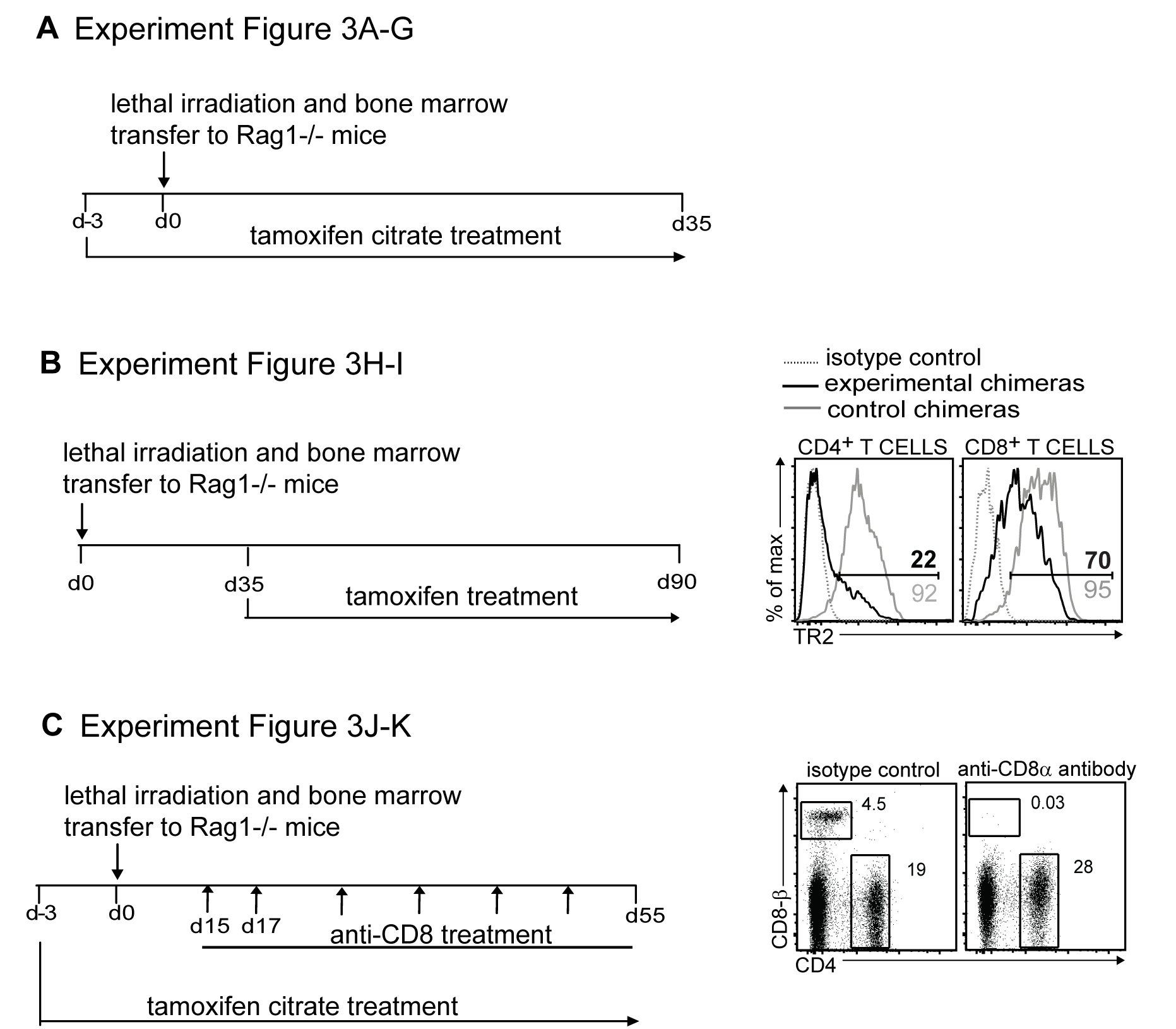

Supplement: Figure S3 — Schemes of experimental setups and FACS analysis of TR2 deletion in respective experimental setups. (A) The scheme of the experiment described in Figure 3A–G. (B) The scheme of the experiment described in Figure 3H. Flow cytometric analysis of TR2 expression by CD4+ and CD8+ T cells from peripheral blood after long-term tamoxifen citrate treatment. (C) The scheme of the experiment described in Figure 3J–K. Flow cytometric analysis of CD4+ and CD8+ T cell frequencies in the spleen of chimeric mice at day 55 following anti-CD8α (YTS 169.4) or isotype control treatment. (TIF) [file pbio.1001674.s003.tif]

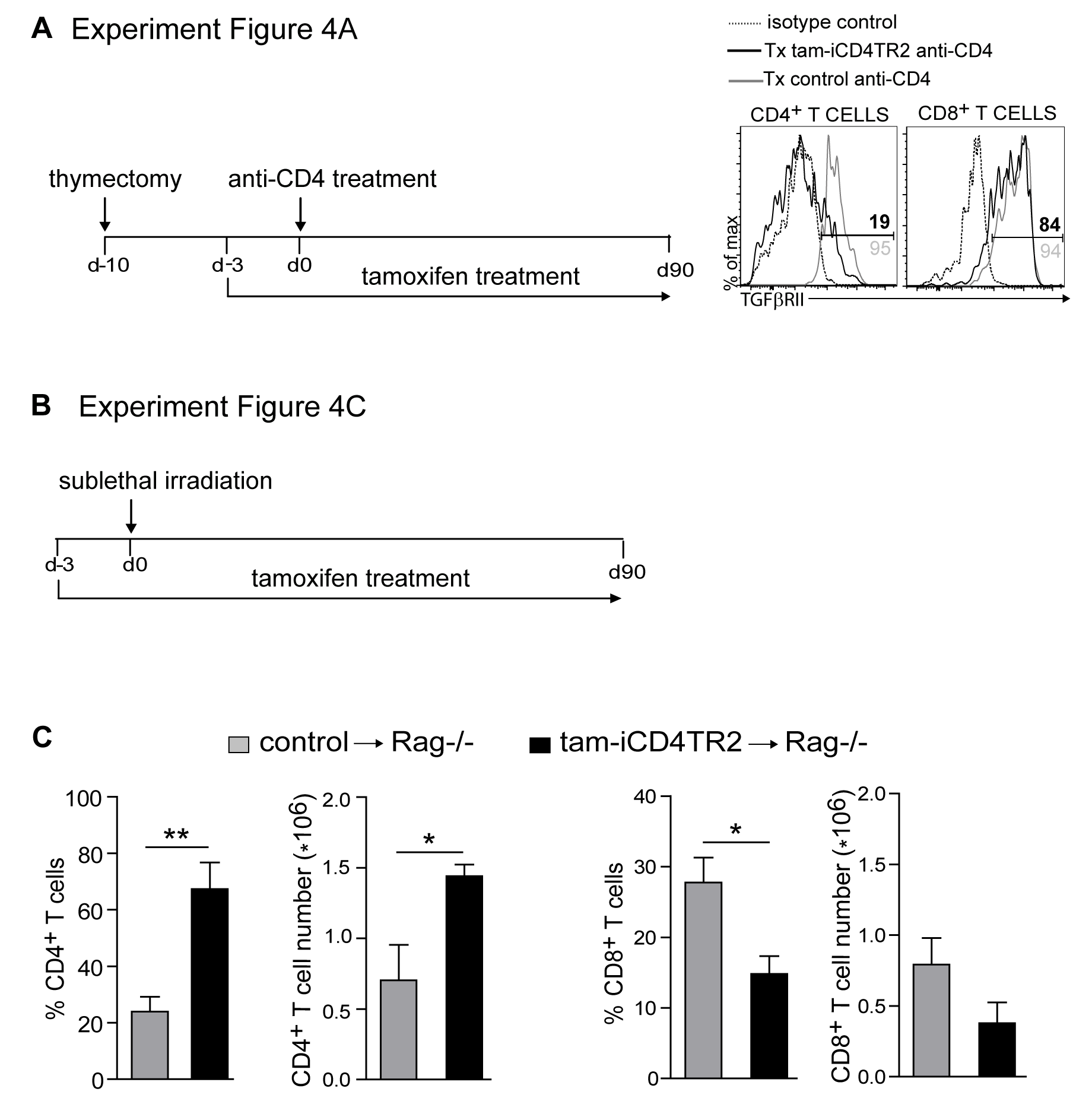

Supplement: Figure S4 — Schemes of experimental setups and FACS analysis of TR2 deletion in lymphopenic environment. (A) Scheme of the experiment described in Figure 4A and flow cytometric analysis of TR2 expression by CD4+ and CD8+ T cells from peripheral blood after long-term tamoxifen citrate treatment (day 90). (B) Scheme of the experiment described in Figure 4C. (C) The percentage and number of CD4+ T cells (left panel) and CD8+ T cells (right panel) in the mesenteric lymph nodes of Rag−/− mice 7 wk after adoptive transfer of tam-iCDTR2 and control T cells (mean ± SEM, 5 mice per group, analysed in two independent experiments). (TIF) [file pbio.1001674.s004.tif]

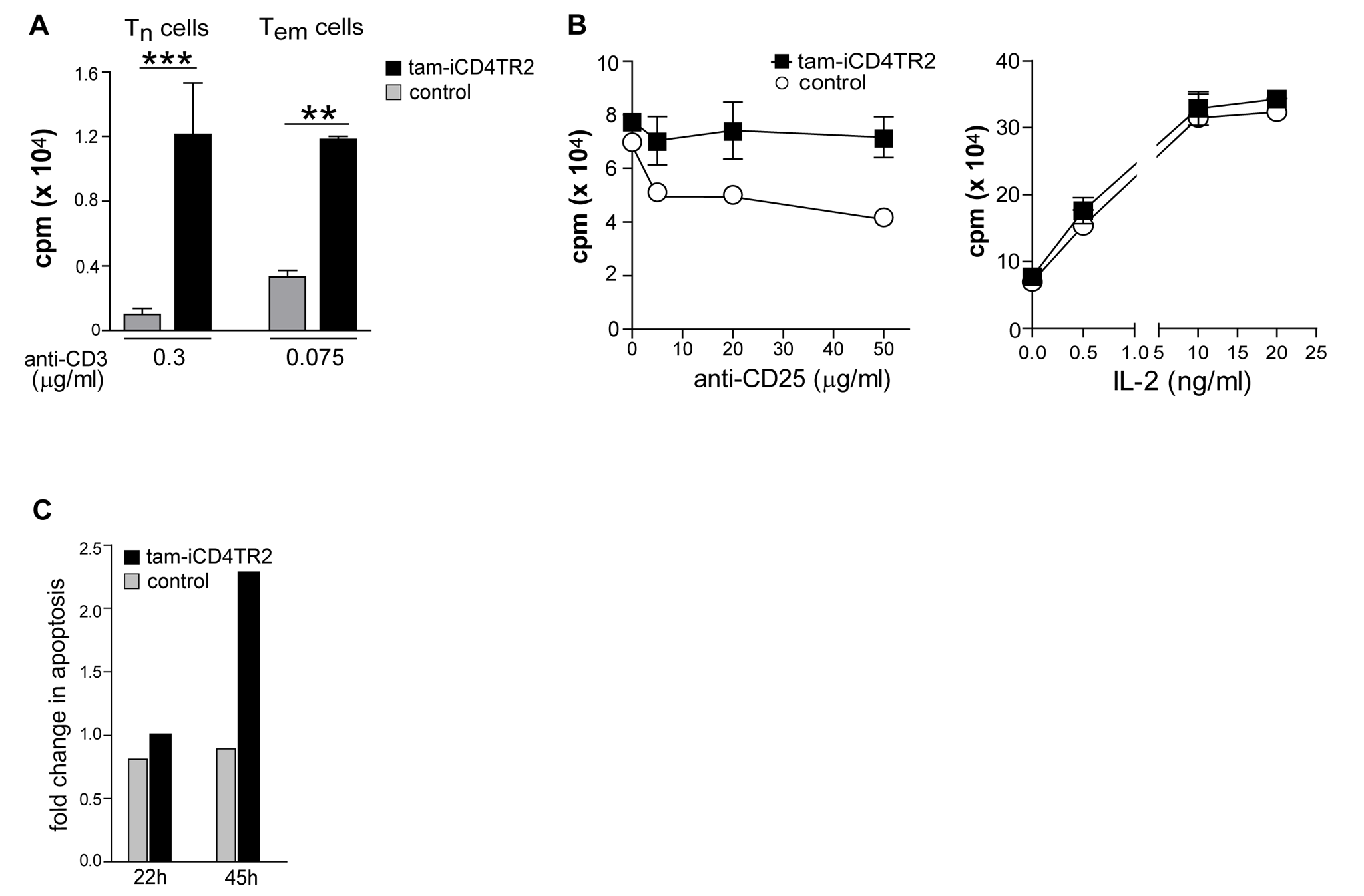

Supplement: Figure S5 — Proliferation of TR2-deficient CD4+ T cells. (A) Sorted effector memory and naïve CD4+CD25− T cells were cultured for 72 h and stimulated with indicated concentrations of anti-CD3 antibody. Thymidine was added for the last 24 h of culture (mean ± SEM, 4 mice per group, analysed in two independent experiments). (B) Proliferation analysis of sorted CD4+ T cells cultured for 72 h with anti-CD3 (0.6 µg/ml) and anti-CD25 (PC61) or with indicated cytokines. Thymidine was added for the last 24 h of culture. (C) In vitro analysis of apoptosis induction. Tam-iCD4TR2 and control cells were cultured in AIM-V medium with or without tamoxifen. The ratio between AnnexinV positive CD4+ T cells that were tamoxifen-treated versus untreated is indicated (mean, 3 mice per group). These data are representative of three independent experiments. (TIF) [file pbio.1001674.s005.tif]

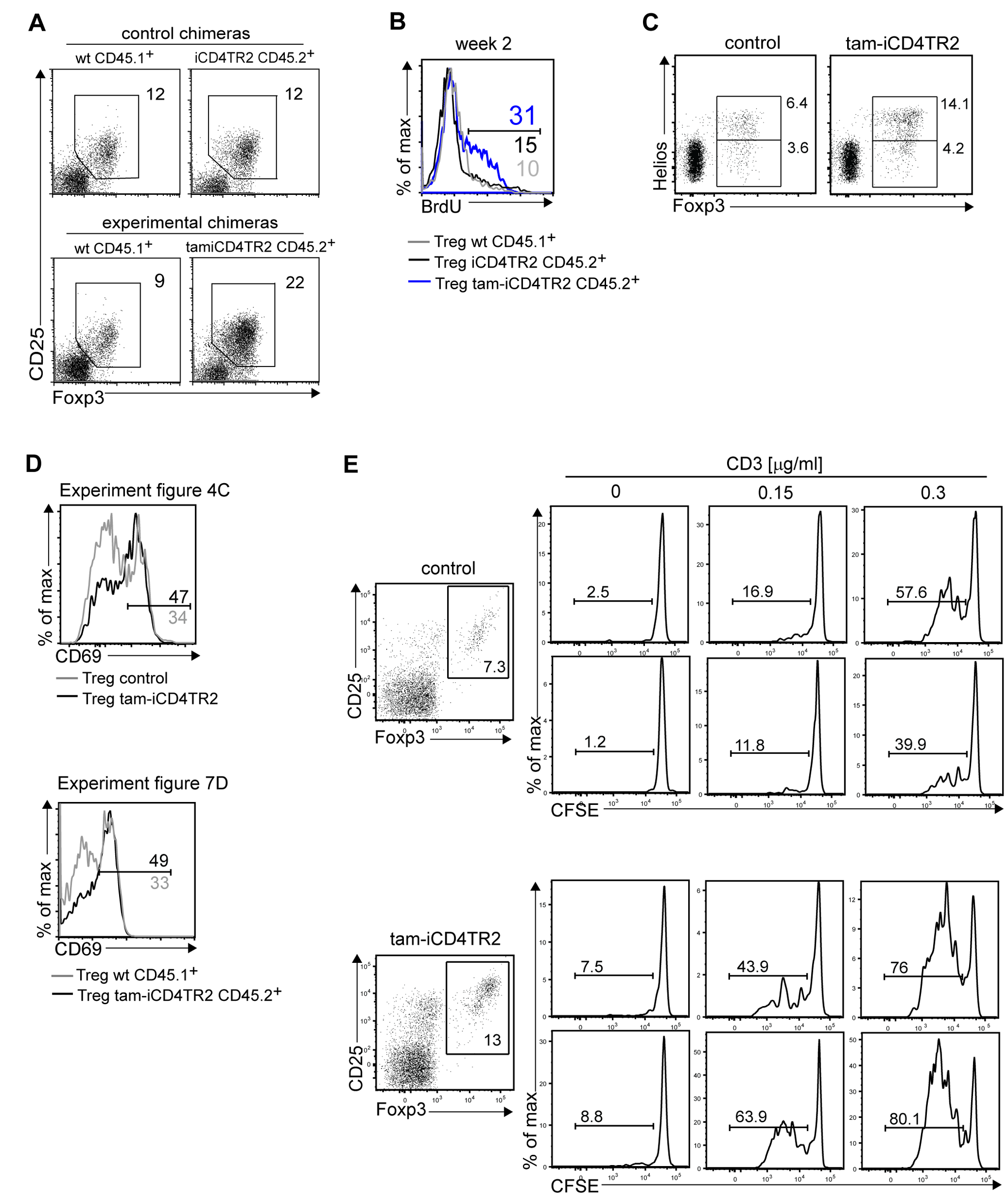

Supplement: Figure S6 — Foxp3 and Helios expression by TR2-deficient regulatory T cells. (A) Flow cytometric analysis of the expression of Foxp3 and CD25 by CD4+ T cells isolated from LN at 2 wk p.a. (B) Flow cytometric analysis of the BrdU positive Treg cells in experimental and control chimeras 2 wk p.a. (C) Flow cytometric analysis of the expression of Helios and Foxp3 by CD4+ T cells isolated from LN at 2 wk p.a. These are representative results of two independent experiments. (D) Flow cytometric analysis of CD69 expression by splenic Treg cells isolated from tam-iCD4TR2 and control mice from indicated experimental setups. (E) Proliferation analysis of Treg cells in vitro. Splenocytes isolated from tami-CD4TR2 and control mice 2 wk p.a. were labelled with CFSE and cultured for 72 h with indicated concentrations of anti-CD3 antibody. Each row represents proliferation of Treg cells from a different mouse. These are representative data of two independent experiments. (TIF) [file pbio.1001674.s006.tif]
